# Risk factors for fatigue severity in the post-COVID-19 condition: A prospective controlled cohort study of nonhospitalised adolescents and young adults

**DOI:** 10.1016/j.bbih.2025.100967

**Published:** 2025-02-18

**Authors:** Joel Selvakumar, Lise Beier Havdal, Elias Myrstad Brodwall, Silke Sommen, Lise Lund Berven, Tonje Stiansen-Sonerud, Erin Cvejic, Vegard Bruun Bratholm Wyller

**Affiliations:** a Department of Paediatrics and Adolescent Health, Akershus University Hospital, PO Box 1000, Lørenskog, N-1478, Norway; b Institute of Clinical Medicine, University of Oslo, PO Box 1171, Blindern, N-0318, Oslo, Norway; c University of Oslo, Oslo, Norway; d Department of Clinical Molecular Biology (EpiGen), University of Oslo and Akershus University Hospital, PO Box 1000, Lørenskog, N-1478, Norway; e The University of Sydney, School of Public Health, Faculty of Medicine and Health, Edward Ford Building (A27) Fisher Road, NSW, 2006, Australia

**Keywords:** Long COVID, Post-COVID-19 condition, Post-acute sequelae of COVID-19, Adolescents, SARS-CoV-2, Post-infective fatigue syndrome, Chronic fatigue syndrome

## Abstract

Long COVID is a global health concern, leading to persistent symptoms and disability long after the acute SARS-CoV-2 infection in most age groups. The condition can manifest even following mild COVID-19, and in young people, it may have serious adverse consequences for educational attainment and transition to adulthood. Fatigue is the most prevalent symptom, but the underlying mechanisms remain poorly understood. In this prospective study of 404 SARS-CoV-2-positive and 105 SARS-CoV-2 negative, non-hospitalised youth (ages 12–25, female 62%), we investigated which factors in the early convalescent stage (<28 days since test) were associated with the severity of persistent fatigue at 6 months after infection. Participants completed questionnaires regarding clinical symptoms, social factors and psychological traits, and were subject to clinical and functional testing and biomarker analyses. Variables with significant (p < 0.2) associations to the outcome in simple linear regression were chosen for multivariable modelling, together with potential confounders. In the final multivariable model, SARS-CoV-2-positivity was a minor risk factor for fatigue severity at six months. Baseline severity of symptoms was the main risk factor and correlated with psychosocial factors such as loneliness and neuroticism, rather than biomarkers. Our results suggest that factors not related to infection are major risk factors for persistent fatigue in this age group.

## 1 Introduction

Post-infective sequelae after acute COVID-19, commonly referred to as ‘Post COVID-19 condition’ (PCC) or ‘Long COVID’, has become a major health challenge worldwide in the aftermath of the COVID-19 pandemic(O'Mahoney et al., 2023). More than 200 symptoms have been described as part of long COVID, however, the most prevalent one across several studies is that of fatigue(Akbarialiabad et al., 2021; Behnood et al., 2022); with a meta-analysis of 54 studies in adults estimating that 0.6–10% of all infected individuals remain fatigued at 3 months post-infection (Global Burden of Disease Long COVID Collaborators, 2022). Despite the high incidence of acute, mild COVID-19 in children and adolescents, most post COVID-19 studies have been conducted on adult populations (Deuel et al., 2022; Mizrahi et al., 2023; Pinto Pereira et al., 2022; Stephenson et al., 2022), with a recent systematic review only finding 11 controlled studies in young people eligible for metanalysis (Behnood et al., 2023).

Post-infective fatigue following SARS-CoV-2 infection has garnered public attention in the wake of the COVID-19 pandemic. However, there exists a substantial body of literature on fatigue following other infectious diseases, such as those caused by Epstein-Barr Virus (EBV), *Borrelia burgdorferi*, SARS-CoV-1 and *Giardia duodenalis* (Hanevik et al., 2014; Jason et al., 2021; Seet et al., 2007). Despite the diversity of the precipitating infective agents, the prevalence of post-infective fatigue has been shown to be similar, with 10–15% experiencing disabling fatigue at six months post-infection (Hickie et al., 2006). Thus, at the very beginning of the pandemic, when most research focused on the acute pathogenicity of SARS-CoV-2, scholars with experience in the field predicted and warned of a subsequent wave of post-infective fatigue (Islam et al., 2020).

Most proposed mechanisms of the pathophysiology of post-COVID-19 symptoms involve factors specific to infection, such as organ-specific tissue-damage, persistent viral reservoirs, autoimmune processes, and reactivation of latent viruses (Altmann et al., 2023; Davis et al., 2023; Douaud et al., 2022; Monje and Iwasaki, 2022; Puntmann et al., 2022; Wu et al., 2024). Some researchers, noting the high prevalence of persistent symptoms in the general population and drawing from research in the pre-COVID-19 era, have proposed a potential role for factors not related to the infection itself; such as learning difficulties, poor social support, negative illness perceptions and negative life events (Bertran et al., 2022; Frontera et al., 2022; Nugawela et al., 2022, 2024; Saunders et al., 2023; Wang et al., 2022). With the intent of investigating factors implicated in both these schools of thought, the aim of the present study is to investigate a broad range of biological, psychological and environmental risk factors for fatigue severity six months after infection. We hypothesised that baseline symptom severity and markers of infection/inflammation severity would be major risk factors for fatigue severity at six months follow-up.

## 2 Materials and methods

### 2.1 Study design

LOTECA (Long-term effects of COVID-19 in Adolescents) was a prospective cohort study of non-hospitalised SARS-CoV-2-positive and -negative adolescents and young adults (ClinicalTrials ID: NCT04686734). The present paper reports one of two primary endpoints of the study. The remaining primary endpoint, as well as a detailed rendition of the methods and materials given below, have been reported elsewhere (Selvakumar et al., 2023).

### 2.2 Participants

Individuals aged 12–25 undergoing SARS-CoV-2 reverse-transcription polymerase chain reaction-testing (RT-PCR) were recruited consecutively from two accredited microbiological laboratories (Fürst Medical Laboratories and Dept. of Microbiology and Infection Control, Akershus University Hospital). Individuals had been tested either due to symptoms, or due to being close contacts of infected individuals. The alpha (B.1.1.7) variant of SARS-CoV-2 was dominant throughout the recruitment period of December 2020 to May 2021. Individuals testing positive were eligible for enrolment after undergoing quarantine (10 days), while individuals testing negative were recruited as controls. Exclusion criteria were: a) greater than 28 days since onset of symptoms or SARS-CoV-2 test; b) hospitalisation due to COVID-19; c) pregnancy; d) serological evidence of previous infection (in the SARS-CoV-2-negative group). The project was approved by the Regional Committee for Ethics in Medical Research (ref. #203645) and written informed consent was obtained as required by the Norwegian Health Research Act. Immunisation history was obtained through linkage with the Norwegian Immunisation Register (Trogstad et al., 2012).

### 2.3 Investigational program

Participants were assessed at baseline and at six months after recruitment at our study centre in Akershus University Hospital, Norway, and underwent a standardised investigational programme consisting of a clinical examination, recording of vital signs, functional testing, blood sampling, and completion of self-report questionnaires.

### 2.4 Functional testing

Spirometry was performed to measure the forced vital capacity (FVC) and the forced expiratory volume in 1 s (FEV1), adhering to the American Thoracic Society and European Respiratory Society guidelines (Graham et al., 2019).

An electrocardiogram was recorded for 5 min and used to calculate indices of heart rate variability (power in the high-frequency [HFRRI], and low-frequency [LF-RRI] range), considered markers of autonomic activity (Task Force of the European Society of Cardiology and the North American Society of Pacing and Electrophysiology, 1996).

Working memory was tested with a digit span test, while the Hopkins Verbal Learning Test-Revised (HVLT-R) was used to assess verbal recognition and recall (Benedict et al., 2010; Grizzle, 2011).

### 2.5 Blood sampling and laboratory assays

Samples were obtained by antecubital venous puncture and routine blood analyses of haematology and biochemistry were carried out (including vitamins B12 and D, cardiac markers, D-dimer and ferritin). In addition, antibodies against SARS-CoV-2 and EBV were analysed by routine methods, and markers of inflammation and immune response were assayed using multiplex technology (Supplemental methods) (Selvakumar et al., 2023; Sommen et al., 2023).

### 2.6 Questionnaires

A questionnaire composed of items from 19 different validated inventories charted comorbidities, current medication, substance abuse, demographic and social variables, clinical symptoms, psychological traits, and quality of life. The endpoint of ‘fatigue’ was charted using the Chalder Fatigue Questionnaire (sum score 0 to 33, where higher scores indicate more fatigue) (Chalder et al., 1993).

### 2.7 Risk factor hypotheses

Potential baseline predictors of persistent symptoms after infection were identified in the literature, and grouped as background/constitutional factors (sex, age, BMI, ethnicity, chronic disorders), observational period characteristics (vaccinations, duration from baseline to follow-up), organ function tests/biomarkers, immunological markers, autonomic markers, cognitive function tests, clinical symptoms, psychological traits, and social/behavioural markers. SARS-CoV-2-status (positive/negative) was considered the independent variable of interest, while background/constitutional factors and observational period characteristics were considered potential confounders.

### 2.8 Statistical analyses

Fatigue score at six months was pre-defined as the primary outcome (Wyller, 2022). Presuming 500 included individuals, the study had a 90% power to detect a variable explaining 2 % of the total variance (R^2^) of the primary outcome (significance level 0.05).

Bivariate analyses between the outcome variable and each independent predictor variable were performed using linear regression. Residual plots were examined, and variables were eventually transformed to meet the assumptions of linear regression. Variables with a p < 0.2 in bivariate analyses were included in multiple linear regression modelling. Preceding multiple linear regression, dimensionality reduction was performed by principal component analysis (PCA) of the variables in the clinical symptoms and psychological traits groups respectively. The PCA-derived components were used instead of the original variables. Similarly, for highly correlated variables (negative life events last year and all negative life events; granulocyte: lymphocyte ratio, systemic inflammation index and lymphocytes; asthma and any comorbidity), the variable with the lowest p-value in bivariate analyses was chosen. Variables were removed in a stepwise manner to obtain a more parsimonious model, until only variables with a p ≤ 0.05 remained. SARS-CoV-2 status, background/constitutional factors and observational period characteristics were included and retained throughout, regardless of the results of bivariate analyses.

**Fig. 1:**
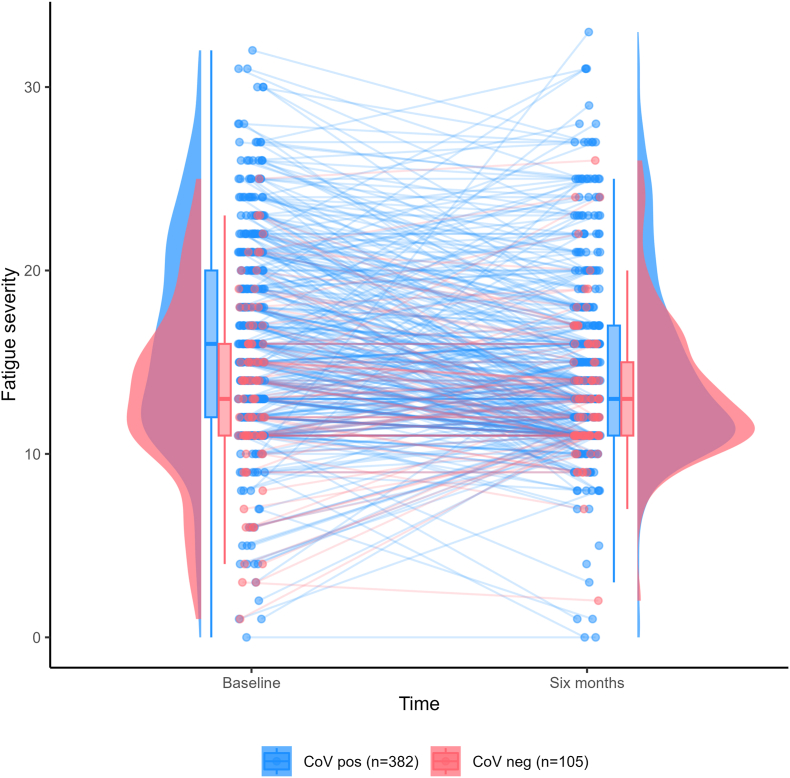
Trajectories of fatigue severity from baseline to six months, n = 467 Caption: Fatigue severity assessed by the Chalder fatigue scale (Chalder et al., 1993), range 0–33, where higher scores imply more severe fatigue. Three participants had missing fatigue scores at baseline.

The analyses above were repeated in two separate datasets as sensitivity analyses: 1) Multiple imputation with predictive mean matching was performed to create 45 imputed datasets, corresponding to the percentage of observations with any missing values, utilising the MICE package (Buuren and Groothuis-Oudshoorn, 2011). PCA was performed on each imputed dataset to create the ‘symptom severity’ and ‘psychological traits’ variables. Bivariate analyses were performed on each individual set and estimates were pooled adhering to Rubin's rules (Buuren, 2018). Multiple linear regression modelling was performed, as described in the previous paragraph, on each set. Variables appearing in at least half of the models (Supplemental Table S3) were included in a full model. Each variable was then removed and a pooled Wald statistic determined whether the variable should be included or not in the final model (Buuren, 2018). 2) To exclude other conditions that may give rise to fatigue, a dataset was created excluding individuals a) with possible EBV infection at inclusion or during the observational period, b) vaccinated before baseline, c) receiving vaccination less than five days prior to the six months assessment, d) with complex chronic conditions (Feudtner et al., 2001), pain-related comorbidities (e.g. migraine) or fatigue-related comorbidities (e.g. chronic fatigue syndrome) *and* evidence of pre-existing fatigue in medical records, or e) with a depression score at baseline of 15 or above (Supplemental Methods). Analyses in this dataset were performed in the same manner as for the main dataset. Statistical analyses were carried out in SPSS version 28.0 (SPSS Inc., Chicago, IL) and R version 4.2.3 (R Foundation for Statistical Computing). Fig. 1 was created using the ggrain package for R (Allen et al., 2021).

## 3 Results

A total of 509 individuals were enrolled in the study, of whom 404 were SARS-CoV-2 positive- and 105 were SARS-CoV-2 negative. Most SARS-CoV-2-positive individuals had been tested due to symptoms, while a minority of 13 individuals (3%) were asymptomatic close-contacts. At six months, 26 participants were lost to follow-up, and additionally 16 participants in the SARS-CoV-2 negative group had evidence of SARS-CoV-2 infection and were excluded. Individuals lost to follow-up had a similar baseline symptom profile as those who attended six month follow-up (Selvakumar et al., 2023). Further details of the recruitment process and source population have been published elsewhere (Selvakumar et al., 2023). Compared to the SARS-CoV-2-negative group, the SARS-CoV-2-positive group had a higher proportion of individuals with non-European ethnicity, a higher proportion in the age group of 21–25 years, and a lower proportion with comorbidities (Table 1).

**Table 1:** Cohort characteristics at baseline and six-month follow-up.

	At inclusion (baseline)		At six-month follow-up	
	*SARS-CoV-2-positive group (n=404)*	*SARS-CoV-2-negative group (n=105)*	*SARS-CoV-2-positive group (n=382)*	*SARS-CoV-2-negative group (n=85)*
**Background**				
Sex – no. males (%)	157 (39)	37 (35)	152 (40)	31 (37)
Age group – no. (%)				
12 < 15	101 (25)	25 (24)	98 (26)	18 (21)
15 < 18	107 (27)	35 (33)	104 (27)	31 (37)
18 < 21	84 (21)	26 (25)	80 (21)	21 (48)
21 < 25	112 (28)	19 (18)	100 (26)	15 (18)
BMI, z-score^a^ – mean (SD)	0.45 (1.2)	0.49 (1.1)	0.52 (1.2)	0.51 (1.1)
95 % CI of the mean	0.34–0.47	0.27–0.70	0.40–0.64	0.27–0.76
Ethnicity				
Non–Caucasian – no. (%)	98 (24)	4 (3.8)	88 (23)	2 (2.4)
Current comorbidity				
Any comorbidity – no. (%)	81 (21)	36 (35)	89 (24)	31 (37)
ADHD – no. (%)	5 (1.3)	3 (2.9)	5 (1.4)	3 (3.6)
Asthma – no. (%)	26 (6.7)	5 (4.8)	27 (7.3)	4 (4.8)
Allergy and atopy – no. (%)	16 (4.1)	10 (9.6)	17 (4.6)	9 (11)
Anxiety and depression – no. (%)	1 (0.3)	3 (2.9)	4 (1.1)	3 (3.6)
Endocrinological – no. (%)	6 (1.5)	1 (1.0)	6 (1.6)	1 (1.2)
Gastrointestinal – no. (%)	5 (1.3)	4 (3.8)	6 (1.6)	5 (6.0)
Gynaecological – no. (%)	4 (1.0)	1 (1.2)	4 (1.0)	1 (1.2)
Neurological including primary headache disorders – no. (%)	10 (2.6)	5 (4.8)	9 (2.5)	4 (2.8)
Socieconomic level				
Parents' highest ISEI–08, score 10–90 – median (IQR)	63 (37, 75)	65 (51, 73)	64 (21)	62 (18)
95 % CI of the median	59–69	60–68	59–69	59–69
Smoking				
Daily – no. (%)	1 (0.3)	0 (0)	1 (0.3)	0 (0)
Not daily – no. (%)	12 (3.1)	3 (2.9)	11 (3.0)	1 (1.2)
Never – no. (%)	376 (97)	101 (97)	355 (97)	84 (99)
COVID-19 immunisation				
No doses – no. (%)	399 (99)	99 (94)	145 (38)	8 (9.4)
One dose – no. (%)	5 (1.2)	4 (3.8)	232 (61)	29 (34)
Two doses – no. (%)	0 (0)	2 (1.9)	5 (1.3)	47 (55)
Three doses – no. (%)	0 (0)	0 (0)	0 (0)	1 (1.2)

**Symptoms and functional impairment**^b^				
Fatigue^c^, score 0 to 33 – median (SD)	16.0 (12.0, 20.0)	13.0 (11.0, 16.0)	13.00 (11.0, 17.0)	12.50 (11.0, 15.0)
95 % CI of the median	15.6–16.8	12.4–14.2	14.0–15.1	12.5–14.1
Post-exertional malaise^d^, score 0 to 100 – median (IQR)	20.0 (5.0–45.0)	10 (1.3–25.0)	10.0 (0–35.0)	10.0 (0–22.5)
95 % CI of the median	15–25	10–15	10–15	5–10
Cognitive symptoms^e^, score 3 to 15 – median (IQR)	6.0 (3.0–8.5)	6.0 (4.3–9.0)	6.0 (4.0–10.0)	6.0 (4.0–8.0)
95 % CI of the median	5–6	5–6	6–7	5–7
Respiratory symptoms^f^, score 2 to 10 – median (IQR)	4.0 (3.0–6.0)	3.0 (2.0–4.0)	3.0 (2.0–5.0)	3.0 (3.0–4.0)
95 % CI of the median	4–5	3–3	3–4	3–4
Symptoms of anxiety^g^, score 0 to 21 – median (IQR)	5.0 (3.0–9.0)	7.0 (4.0–10.0)	6.0 (3.0–9.0)	5.0 (3.5–10.0)
95 % CI of the median	5–6	6–8	5–6	5–7
Symptoms of depression^h^, score 0 to 21 – median (IQR)	3.0 (1.0–6.0)	3.5 (2.0–6.0)	3.0 (1.0–6.0)	3.0 (1.0–7.0)
95 % CI of the median	3–4	3–5	2–3	2–5
Quality of life^i^, score 0 to 100 – median (IQR)	77.2 (63.6–88.0)	77.2 (65.2–84.8)	78.3 (66.3–88.0)	76.1 (67.9–86.4)
95 % CI of the median	75–80	72–79	77–80	72–79

**Clinical findings**				
Days since symptom onset/PCR test – median (IQR)	18 (15–21)	17 (14–21)	213 (207–224)	210.0 (205–218)
Range	8–28	2–27	170–341	195–243
Time span between baseline and follow-up, days – median (IQR)	NA	NA	193.0 (188.0–205.0)	193 (188.0–200.0)
Range	NA	NA	164–326	183–252
Tympanic temperature, ^o^C – mean (SD)	36.8 (0.4)	36.7 (0.4)	36.6 (0.4)	36.7 (0.4)
95 % CI of the mean	36.7–36.8	36.6–36.7	36.6–36.7	36.6–36.7
Respiratory frequency, breath/min – mean (SD)	16.7 (4.2)	16.6 (3.7)	15.3 (3.2)	15.1 (3.2)
95 % CI of the mean	16.2–17.1	15.9–17.3	15.0–15.6	14.4–15.8
SpO_2_, % – mean (SD)	98.6 (1.1)	98.6 (1.2)	98.5 (1.1)	98.3 (1.3)
95 % CI of the mean	98.5–98.8	98.3–98.8	98.4–98.7	98.0–98.6
FEV1:FVC ratio – mean (SD)	0.86 (0.07)	0.86 (0.07)	0.85 (0.07)	0.86 (0.06)
95 % CI of the mean	0.85–0.86	0.85–0.87	0.84–0.85	0.84–0.87
FVC, % of predicted^j^ – mean (SD)	99.5 (10.0)	100.4 (10.3)	99.5 (10.3)	99.9 (9.9)
95 % CI of the mean	98.4–100.6	98.3–102.5	98.4–100.6	97.7–102.2

**Laboratory findings**				
Blood Haemoglobin, g/dL – mean (SD)	13.5 (1.2)	13.5 (1.1)	13.6 (1.2)	13.7 (1.0)
95 % CI of the mean	13.4–13.6	13.3–13.8	13.5–13.7	13.4–13.9
Blood Platelet count, 10^9^ cells/L – mean (SD)	260 (57)	254 (51)	270 (59)	276 (58)
95 % CI of the mean	254–266	244–265	264–276	263–289
Blood Leukocyte count, 10^9^ cells/L – mean (SD)	5.9 (1.5)	5.6 (1.3)	6.1 (1.8)	5.9 (1.5)
95 % CI of the mean	5.8–6.1	5.4–5.9	5.9–6.3	5.5–6.2
Plasma hsCRP, mg/L – median (IQR)	0.8 (0.4–2.6)	1.3 (0.5–3.5)	1.3 (0.45–4.24)	1.8 (0.7–5.7)
95 % CI of the median	0.7–1.1	0.7–1.7	1.0–1.6	1.0–3.2
Plasma ferritin, μg/L – median (IQR)	69 (43–107)	48 (33–71)	45 (30–77)	44 (33–63)
95 % CI of the median	64–76	45–56	40–50	40–54
Plasma creatinine, mmol/L – mean (SD)	62 (13)	61 (12)	68 (13)	68 (12)
95 % CI of the mean	61–63	59–64	66–69	66–71
Plasma ALT, U/L – median (IQR)	16.0 (11.0, 22.0)	15.0 (12.0, 20.0)	17.0 (13.0, 23.0)	16.0 (13.0, 20.0)
95 % CI of the median	15–16	14–18	16–18	15–18
HbA1c, mean (SD)	34.0 (3.4)	31.9 (2.9)	33.0 (3.4)	32.4 (2.8)
95 % CI of the mean	33.7 - 34.4	31.3 - 32.5	32.7 - 33.4	31.8 - 33.0
Plasma D-dimer, mg/L – median (IQR)	0.18 (0.08, 0.27)	0.19 (0.12, 0.27)	0.13 (0.07, 0.20)	0.15 (0.06, 0.20)
95 % CI of the median	0.2–0.2	0.2–0.2	0.1–0.1	0.1–0.2
Serum NT-proBNP, ng/L – median (IQR)	34.5 (21.3, 57.0)	34.0 (21.0, 54.0)	30.0 (19.0, 49.0)	34.0 (20.0, 54.0)
95 % CI of the median	31–38	26–37	28–34	26–42
Serum Troponin T, ng/L – median (IQR)	4.0 (2.4, 6.0)	4.0 (1.6, 5.0)	2.1 (1.1, 4.0)	2.1 (0.8, 4.0)
95 % CI of the median	4.0–4.0	2.6–4.0	1.9–2.3	1.4–2.8

Plasma IL-1β, pg/mL – median (IQR)	0.63 (0.01, 0.98)	0.01 (0.00, 0.22)	NA^l^	NA^l^
	0.47-0.73	0.01-0.19		
Plasma IL-2, pg/mL – median (IQR)	0.69 (0.02, 1.77)	0.03 (0.02, 1.66)	NA^l^	NA^l^
95 % CI of the median	0.47–0.81	0.03–0.78		
Plasma IL-4, pg/mL – median (IQR)	1.44 (1.07, 1.87)	0.88 (0.67, 1.35)	1.07 (0.22, 1.95)	0.26 (0.16, 1.17)
95 % CI of the median	1.33–1.50	0.75–0.88	1.03–1.17	0.21–0.38
Plasma IL-7, pg/mL – median (IQR)	12.56 (4.99, 18.74)	3.90 (1.79, 12.19)	NA^l^	NA^l^
95 % CI of the median	11.46–12.56	2.05–5.65		
Plasma IL-8, pg/mL – median (IQR)	0.80 (0.12, 2.15)	0.10 (0.04, 0.22)	NA^l^	NA^l^
95 % CI of the median	0.58–1.08	0.08–0.12		
Plasma IL-9, pg/mL – median (IQR)	67.4 (27.2, 157.2)	69.7 (39.6, 154.6)	96.5 (50.5, 198.8)	74.8 (40.0, 161.7)
95 % CI of the median	60.5–80.2	55.9–83.1	84.7–108.7	62.2–95.5
Plasma IL-12p70, pg/mL – median (IQR)	1.49 (0.21, 4.84)	0.19 (0.09, 2.38)	NA^l^	NA^l^
95 % CI of the median	1.38–1.50	0.15–0.26		
Plasma IL-13, pg/mL – median (IQR)	0.26 (0.02, 0.58)	0.56 (0.25, 1.17)	0.22 (0.12, 1.58)	0.23 (0.11, 1.23)
95 % CI of the median	0.25–0.27	0.45–0.66	0.17–0.53	0.16–0.71
Plasma IL-17A, pg/mL – median (IQR)	1.62 (0.24, 2.98)	1.35 (0.35, 2.72)	NA^l^	NA^l^
95 % CI of the median	1.31–1.99	0.69–2.03		
Plasma C3bc – median (IQR)	3.87 (2.75, 5.00)	2.95 (2.50, 3.74)	3.71 (3.00, 4.59)	3.35 (2.55, 4.14)
95 % CI of the median	3.68–4.15	2.79–3.15	3.54–3.83	2.70–3.15
Plasma TCC/C5b-9, CAU/mL – median (IQR)	0.18 (0.09, 0.29)	0.003 (0.002, 0.16)	0.19 (0.13, 0.26)	0.18 (0.10, 0.28)
95 % CI of the median	0.17–0.20	0.002–0.05	0.18–0.20	0.15–0.23
MCP-1/CCL2, pg/mL – mean (CI)	12.3 (8.8, 16.1)	12.5 (9.7, 18.0)	5.2 (3.3, 7.0)	2.2 (0.3, 4.5)
95 % CI of the mean	11.9–12.9	11.7–14.0	4.6–5.3	1.7–3.3
MIP-1α/CCL3 – median (IQR)	0.77 (0.56, 0.96)	0.86 (0.64, 1.02)	0.21 (0.04, 0.80)	0.04 (0.02, 0.64)
95 % CI of the median	0.67–0.82	0.79–0.92	0.19–0.31	0.03–0.19
MIP-1β/CCL4 – median (IQR)	24.7 (12.0, 49.8)	24.9 (15.6, 46.3)	27.5 (16.5, 52.7)	23.6 (12.3, 40.8)
95 % CI of the median	22.5–27.2	20.7–27.8	24.8–31.5	16.1–28.2
RANTES/CCL5, pg/mL – median (IQR)	266 (120, 522)	266 (181, 482)	140 (91, 259)	121 (75, 222)
95 % CI of the median	237–295	230–310	130–153	95–144
Plasma IP-10, pg/mL – median (IQR)	149.1 (115.4, 186.5)	116.6 (98.6, 147.5)	106.2 (80.4, 145.9)	90.8 (70.0, 129.2)
95 % CI of the median	142.0–155.5	106.3–123.3	101.7–112.3	81.8–103.5
Plasma GDF15, ng/m – median (IQR)	0.37 (0.31, 0.45)	0.36 (0.30, 0.45)	0.43 (0.36, 0.50)	0.41 (0.36, 0.48)
95 % CI of the median	0.36-0.38	0.33-0.39	0.42-0.45	0.40-0.44
Plasma Interferon γ,^–^median (IQR)	1.22 (0.40, 1.88)	0.94 (0.52, 1.76)	NA^l^	NA^l^
95 % CI of the median	1.02–1.34	0.94–1.14	NA^l^	NA^l^
Plasma GM-CSF – median (IQR)	0.11 (0.02, 0.60)	0.02 (0.01, 0.03)		
95 % CI of the median	0.11–0.34	0.01–0.02	NA^l^	NA^l^
Serum SARS-CoV-2 antibody titer^k^ – median (IQR)	4.0 (14)	0 (0)	23 (44)	0.1 (0.01)
95 % CI of the median	3.0–5.6	0–0	20–26	0.1–0.1
SARS-CoV-2-Anti-RBD, BAU/mL – median (IQR)	1044 (1690)	1 (0)	5628 (11566)	3508 (8284)
95 % CI of the median	986–1131	1–1	5063–6424	1894–5392

SARS-CoV-2= Severe acute respiratory syndrome coronavirus 2; SD=Standard deviation; IQR = interquartile range; CI=Confidence interval; PCR=Polymerase chain reaction; NA=Not Applicable; BMI=Body mass index; ISEI=International socio-economic index; FEV1=Forced Expiratory Volume of 1st second; FVC=Forced Vital Capacity; SpO_2_=Peripheral oxygen saturation; hsCRP = high-sensitive assay of C-reactive protein; ALT = Alanine aminotransferase; NT-proBNP=N-terminal pro-Brain Natriuretic Peptide.

a Standardised score calculated according to World Health Organization (2006) Child Growth Standards.

b With the exception of ‘Quality of life’, higher values imply more symptoms. For ‘Quality of life’, higher values imply higher quality of life and less functional impairment.

c From the Chalder Fatigue Questionnaire.

d From the DePaul Symptom Questionnaire.

e The sum score across the three items “memory problems”, “concentration problems” and “decision making problems”.

f The sum score across the two items “dyspnoea” and “coughing”.

g From the Hospital Anxiety and Depression Scale; anxiety subscale.

h From the Hospital Anxiety and Depression Scale; depression subscale.

i From the Pediatric Quality of Life Inventory.

j The Global Lung Function Initiative 2012 reference values were used to calculate predicted values.

k Total anti-nucleocapsid IgG and IgM.

l Not analysed at 6 months, because of too many values below detection limit and/or missing.

SARS-CoV-2-positive individuals were more severely fatigued compared to -negative individuals at baseline, though median severity was similar at six months. However, the distribution was right-skewed and a greater proportion of SARS-CoV-2-positive individuals had severity scores above the upper quartile (Fig. 1).

The average missingness across all variables was 3%, and 307 participants had data available for all variables (Supplemental Table S1).

**Fig. 2:**
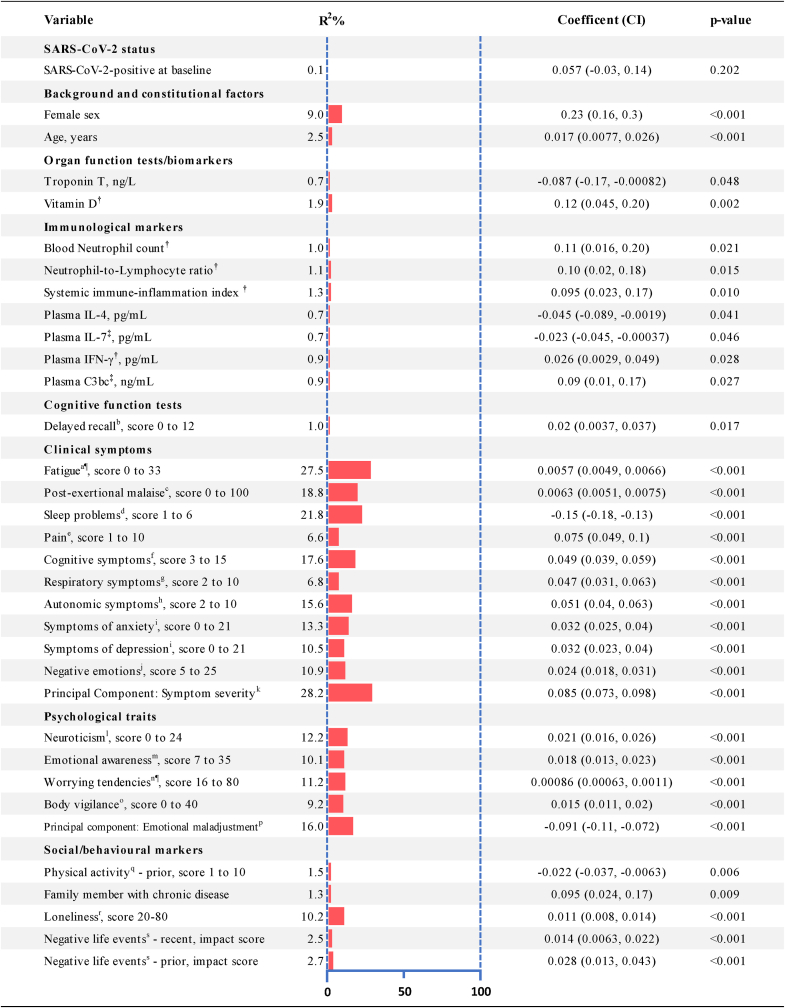
Baseline risk factors and their univariate associations to fatigue severity^a^∗. Caption: Linear regression. Only associations with p < 0.5 are shown for brevity. Values for all variables are given in Supplemental table S5. Footnotes: BMI=Body mass index; CI = 95% Confidence interval; SARS-CoV-2= Severe acute respiratory syndrome coronavirus 2; FVC=Forced vital capacity; IL=Interleukin. ^a^As measured by the Chalder Fatigue Questionnaire, score 0–33, higher scores imply more fatigue. ^b^From the Hopkins Verbal Learning Test revised (HVLT-R); higher scores imply better delayed recall of words. ^c^From the DePaul Symptom Questionnaire; higher score implies more frequent post-exertional malaise. ^d^From the Karolinska Sleep Questionnaire; higher score implies better sleep. ^e^From the Brief Pain Inventory, higher score implies more pain. ^f^Self-developed, aggregated score for problems with ‘memory’, ‘concentration’, and ‘decision making’; higher score implies more symptoms. ^g^Self-developed, aggregated score for symptoms ‘cough’ and ‘dyspnoea’; higher score implies more symptoms. ^h^Self-developed, aggregated score for symptoms ‘dizziness’, ‘cold and pale hands’, ‘feeling alternately warm and cold’; higher score implies more symptoms. ^i^From the anxiety and depression subscales, respectively, of the Hospital Anxiety and Depression Scale; higher scores imply more symptoms. ^j^From the Positive and Negative Affect Schedule; higher score implies more negative emotions. ^k^The main component extracted by Principal Component Analysis of the 10 clinical symptoms variables, labelled ‘symptom severity’. ^l^From the NEO-Five-Factor-Inventory-30; higher scores implies more neuroticism. ^m^From the Toronto Alexithymia Scale; higher score implies more difficulty identifying feelings. ^n^From the Penn State Worry Questionnaire; higher score implies more worrying. ^o^From the Body Vigilance Scale; higher score implies being more attentive to bodily sensations. ^p^The main component extracted by Principal Component Analysis of the four psychological traits variables, labelled ‘emotional maladjustment’. ^q^Self-developed; higher score implies more physical activity. ^r^From the University of California, Los Angeles, Loneliness Scale; higher score implies more loneliness. ^o^From the Life Event Checklist; higher score implies more negative impact of past life events. ∗ ln(x+1), † natural logarithm, ‡square-root, § Cube root, and ¶ fifth root transformations were applied to respective variables for regression analyses.

In bivariate analyses (Fig. 2, Supplemental Table S4), SARS-CoV-2 was not a risk factor of six-month fatigue severity. All baseline symptoms were strong risk factors, most notably that of fatigue (coefficient of determination (R^2^) 27.5%; regression coefficient (*B*) 0.0057, 95% confidence interval (CI) 0.0049 to 0.0066). Psychological traits were moderate risk factors, particularly neuroticism (R^2^ 12.2%; *B* 0.021, CI 0.016 to 0.026) and worrying tendencies (R^2^ 11.2%; *B* 0.00086, CI 0.00063 to 0.0011). Performing PCA on ‘clinical symptoms’ and ‘psychological traits’-variables derived one principal component each (Supplementary Table S2), labelled respectively ‘symptom severity’ and ‘emotional maladjustment’. Both components were strong risk factors of fatigue severity at six months, as well as correlated highly with each other (Supplemental Fig. S1). Several social/behavioural factors were also associated, with loneliness in particular a strong risk factor (R^2^ 10.2%; *B* 0.9, CI 0.11 to 0.07). Female sex (R^2^ 9.0 %; *B* 0.23, CI 0.16 to 0.3; p < 0.001) and age (R^2^ 2.5%; *B* 0.02, CI 0.01 to 0.03; p < 0.001) were the only constitutional factors associated with the outcome. Several biomarkers had weak, but significant, associations with fatigue severity.

In multivariable regression, the final model retained baseline symptom severity as the main risk factor (Table 2), as well as the baseline inflammatory markers interleukin (IL)-4, IL-7 and C3bc (the activation product of complement C3). SARS-CoV-2 was a significant independent variable in the final model. Post-hoc analyses showed that in models controlling for higher-order confounders only, SARS-CoV-2 status remained significant (ΔR^2^ 0.6%; *B* 0.09, CI 0.003 to 0.2); p = 0.04), while IL-4, IL-7, and C3bc did not. This could imply that the latter three's inclusion in the final multivariable model could be due to residual confounding from the symptom severity variable. In sensitivity analyses, results were comparable to those of the main analyses (Supplemental Tables S5, S6, S7 and S8).

**Table 2:** Baseline independent predictors of fatigue severity^a^ at six months follow-up (per protocol data). Final multiple linear regression model.

	*Coefficient (CI)*	p-value	Δ *R*^*2*^*(%)*
Intercept	2.649	<0.001	NA
**SARS-CoV-2 status**			
SARS-CoV-2-positive at baseline	0.109 (0.029–0.190)	0.008	1.1
**Background and constitutional factors**			
Female sex	0.113 (0.050–0.176)	<0.001	1.9
Age, years	0.004 (−0.004 to 0.013)	0.333	0.1
BMI, z-score^b^	−0.018 (−0.045 to 0.008)	0.180	0.3
Ethnicity non-European	−0.0751 (−0.150 to 0.0002)	0.051	0.6
Any comorbidity	0.005 (−0.065 to 0.074)	0.890	0.003
**Observational period characteristics**			
Time span between baseline and follow-up, days	−0.001 (−0.003 to 0.001)	0.228	0.2
Immunisation against SARS-CoV-2^c^	0.240 (0.008–0.471)	0.043	0.6
**Remaining predictor variables**			
Plasma Interleukin-4, pg/mL	−0.051 (−0.091 to −0.011)	0.012	1.0
Plasma Interleukin-7, pg/mL^d^	−0.0211 (−0.042 to −0.001)	0.043	0.6
Plasma C3bc, ng/mL^d^	0.076 (0.006–0.146)	0.035	0.7
Principal component: Symptom severity^e^	0.081 (0.068–0.094)	<0.001	21.9

Adjusted R^2^ for the full model was 34.1%. N = 434 observations. F-statistic 19.7, p-value <0.001.

CI = 95% Confidence interval; NA=Not applicable; SARS-CoV-2= Severe acute respiratory syndrome coronavirus 2; BMI=Body mass index; SE = Standard error.

a As assessed by the Chalder Fatigue Questionnaire, score 0–33, higher scores imply more fatigue. Loge(x+1) transformation was used for regression analyses.

b Standardised score calculated according to World Health Organisation (2006) Child Growth Standards for ages 12–19; for participants above this age, reference values for 19-year-olds were used.

c One or more doses of immunisation against SARS-CoV-2.

d Square root-transformed variable was used for regression analyses.

e The main component extracted by Principal Component Analysis of the 10 clinical symptoms variables, labelled ‘symptom severity’.

## 4 Discussion

The main results from the present study were; (1) SARS-CoV-2 infection was a statistically significant risk factor for fatigue severity at six months, however explained only a small portion of the variance and thus was a minor risk factor(2) Symptom severity at baseline was the strongest risk factor for fatigue severity at 6 months; (3) Psychological, behavioural, and environmental factors were also associated with fatigue at 6 months.

In our previous work from the present cohort (Selvakumar et al., 2023), we found that SARS-CoV-2 was not a risk factor for Post COVID-19 condition, i.e. that due to a high prevalence of symptoms in the SARS-CoV-2 negative group, the proportion adhering to the WHO symptom criteria for PCC was similar among infected and non-infected adolescents. Similarly, although absolute estimates of prevalence differ, other studies in adolescents also find high symptom prevalence in their control groups (Berg et al., 2022; Gross et al., 2024; Pinto Pereira et al., 2022). However, most studies use a dichotomous ‘yes’/‘no’ of symptom presence, that might not capture the difference in severity between infected- and non-infected groups. The novelty of the current study is that rather than using a dichotomous outcome, the addition of a measure of severity increases the relevance of SARS-CoV-2 as a risk factor. This reflects the distribution of fatigue severity at six months (Fig. 1): While the median severity remains similar between groups, a greater proportion of SARS-CoV-2-positive individuals remain in the upper quartile. However, even with the use of a measure for outcome severity, infection with SARS-CoV-2 remained a minor risk factor and explains only a small fraction of the variance. Thus, although incorporating severity criteria could improve the specificity of future long COVID case definitions, our findings suggest that fatigue unrelated to infection remains a significant concern in this age group.

Baseline symptom severity, the major risk factor in the current study, interestingly correlated only weakly with SARS-CoV-2 status. Rather, it correlated with psychosocial factors not specific to infection (Supplemental Fig. S1), such as personality traits, loneliness, and recent negative life events. Again, this reflects the findings from several studies of a high background prevalence of symptoms in this age group during the pandemic, irrespective of infection status (Berg et al., 2022). Notably, in the largest longitudinal study of adolescents to date, *Pinto*-*Pereira* et al. found that fatigue appeared and waned at similar rates in both SARS-CoV-2 positive and -negative participants in the follow-up period after infection, suggesting a role of factors not related to infection (Pinto Pereira et al., 2022; Stephenson et al., 2023).

Research on the relationship between personality and post-COVID-19 symptoms is scarce, however it has previously been explored in a Dutch, longitudinal cohort: Utilising pre-pandemic questionnaire data as predictors, *Slurink* et al. found that neuroticism, agreeableness, low general mood and negative affect were predictive of persistent symptoms in SARS-CoV-2-positive-positive individuals (Slurink et al., 2024). Such associations are also found in the general population, with a meta-analysis of seven prospective studies finding that neuroticism was associated with incident fatigue at later timepoints (Stephan et al., 2022). Though intriguing, it should be noted that personality traits explained a modest portion of the variance of fatigue in the present study, and thus a low score on e.g. neuroticism did not preclude persistent fatigue, nor did a high score implicate fatigue.

Loneliness is associated with a number of adverse cardiovascular, metabolic and developmental health outcomes (Almeida et al., 2021), and has been increasingly recognised as a determinant of health in its own right (O'Sullivan et al., 2022). Of relevance, in a large study categorising the loneliness trajectories of 5851 SARS-CoV-2-positive and -negative adolescents, *Schneider* et al. reported that individuals with ‘high loneliness’ had 16 times the odds of persistent symptoms compared to those with ‘no loneliness’, even while adjusting for baseline loneliness and health (Schneider et al., 2023). The prospect that loneliness and other adverse social factors, worsened by the pandemic (Ernst et al., 2022), could impact persistent symptoms and disability warrants further investigation regarding causality and possible mechanisms.

As mentioned above, baseline psychosocial variables (e.g. emotional maladjustment, loneliness) and baseline symptom severity variables correlated with the outcome, but also with each other. Since both groups of variables were recorded at the same timepoint in the present study (after infection/PCR-testing), we cannot readily infer that the former are a cause of the latter. However, studies examining risk factors *prior* to infection have also implicated psychosocial factors (Matta et al., 2023; Reme et al., 2023; Slurink et al., 2024; Thompson et al., 2022; Tsampasian et al., 2023; Wang et al., 2022): For instance, a large, French longitudinal study found depressive symptoms at the beginning of the pandemic to be associated with a near threefold increase in the odds of persistent symptoms in both SARS-CoV-2 positive and SARS-CoV-2 negative participants(Matta et al., 2023). Similarly, a meta-analysis of 10 UK longitudinal studies found pre-pandemic psychological distress to be one of the strongest health factors associated with symptoms lasting more than 12 weeks (Thompson et al., 2022). Correspondingly, Hartung et al. found pre-infection neuropsychiatric disease (mainly depression, migraine, anxiety), as well as depressive symptoms and anxiety at 6 months, to be significant predictors of *non-recovery* from fatigue at 18 months after infection (Hartung et al., 2024). Perhaps complementary to such reports, a randomised, controlled study of cognitive behavioural therapy found a positive effect on post-COVID-19 fatigue (Cohen's d = 0.69), that was sustained at 12 months (Kuut et al., 2023).

The strengths of our study include a concurrent PCR- and antibody-negative control group; the assessment of clinical, biological and self-report data; and a low drop-out rate. Our study has several limitations. First, as with other community-based observational studies, it is prone to self-selection bias. Individuals recruited to the study, might have been more vulnerable to non-specific stressors compared to the source population. Second, the present infection (upon inclusion) was the first known infection of the individuals in the SARS-CoV-2 positive group. However, we cannot rule out that some unknowingly might have had prior infections, or that some might have had a reinfection between baseline and six-month follow-up, affecting symptom prevalence in the study. However, the low incidence of SARS-CoV-2-infection in Norway pre-2022 makes it unlikely that this would affect our results considerably(Tunheim et al., 2024). Third, the modest size of the control group reduced statistical power. Fourth, although our analyses did not find a role for immunological factors, subgrouping according to phenotype might reveal other characteristics (Sommen et al., 2023). Similarly, the present study includes participants across a wide range of ages (12–25), with individuals near either end of the spectrum facing different biological and environmental circumstances. Subgrouping according to age might reveal other associations. Finally, the study was conducted in a mainly non-vaccinated, immune-naïve sample, at a time when adolescents were potentially exposed to adverse effects of public health measures and other pandemic-related stressors. These are factors potentially increasing symptom prevalence and severity in our sample, compared to individuals in the present era (Atchison et al., 2023).

Taken together, our study found that SARS-CoV-2 was a minor risk factor for post-COVID-19 fatigue. Baseline symptom severity was the main risk factor, and correlated with social and psychological factors, rather than markers of infection and immune activation. Our findings underscore the need for a broad, multidisciplinary approach in the research and treatment of post-COVID-19 fatigue.

## CRediT authorship contribution statement

**Joel Selvakumar:** Writing – review & editing, Writing – original draft, Visualization, Project administration, Investigation, Formal analysis, Data curation. **Lise Beier Havdal:** Writing – review & editing, Investigation, Data curation. **Elias Myrstad Brodwall:** Writing – review & editing, Investigation. **Silke Sommen:** Writing – review & editing, Investigation, Data curation. **Lise Lund Berven:** Writing – review & editing, Resources, Investigation, Data curation. **Tonje Stiansen-Sonerud:** Writing – review & editing, Resources, Investigation. **Erin Cvejic:** Writing – review & editing, Supervision, Methodology. **Vegard Bruun Bratholm Wyller:** Writing – review & editing, Supervision, Resources, Project administration, Methodology, Funding acquisition, Data curation, Conceptualization.

## Data sharing

Anonymised individual participant data as well as data dictionary will be made available with publication for scientific purposes upon reasonable request. The Services for Sensitive Data at the University of Oslo will be used as the data sharing platform (https://www.uio.no/english/services/it/research/sensitive-data/index.html); access to the designated server area will be granted by the VBBW (email: v.b.b.wyller@medisin.uio.no). The study protocol (encompassing the informed consent form) as well as the statistical analysis plan is freely available from the designated ClinicalTrial registry website (https://www.clinicaltrials.gov/ct2/show/NCT04686734).

## Funding

The 10.13039/501100005416Norwegian Research Council [grant #302079], and the 10.13039/501100011761DAM foundation [grant #2022/F0387180], as well as institutional support from Dept. of Paediatrics and Adolescent Medicine, 10.13039/501100012446Akershus University Hospital, and Institute of Clinical Medicine, 10.13039/501100005366University of Oslo.

## Declaration of competing interest

The authors declare that they have no known competing financial interests or personal relationships that could have appeared to influence the work reported in this paper.

## Data Availability

Anonymised individual participant data as well as data dictionary will be made available with publication for scientific purposes upon reasonable request to VBBW; through a dedicated sharing platform.
