# Supplementary material for: Risk factors for fatigue severity in the post-COVID-19 condition: A prospective controlled cohort study of nonhospitalised adolescents and young adults
Source: Brain Behav Immun Health. 2025 Feb 18;44:100967. doi: 10.1016/j.bbih.2025.100967 (PMC11908541; doi:10.1016/j.bbih.2025.100967)
Supplement: Multimedia component 1 [file mmc1.docx]

**Supplementary material**

**Contents**

| **1. Materials and methods – supplementary**  Statistical analyses | |  |
| --- | --- | --- |
|  |  |  |
| **2. Results – supplementary**  **Table S1.** Analyses of missing data. Characteristics of independent variables and their association to complete cases at six months follow-up.  **Table S2.** Results of final factor analyses (Principal Component Analysis) of ten ‘clinical symptoms’ variables and four ‘psychological traits’ variables, respectively. Per protocol data.  **Table S3.** Baseline independent variables associated with fatigue severity at six months follow-up. Overview of results from final multiple linear regression models in 45 imputed sets^a^.  **Table S4**. Characteristics of potential baseline predictors and their univariate associations (linear regression) to fatigue severity at 6 month follow-up. Per protocol data  **Table S5**. Baseline predictors and their univariate associations (linear regression) to fatigue severity^a^ at 6 month follow-up. Sensitivity analysis, sensitivity dataset #1  **Table S6.** Baseline predictors and their univariate associations (linear regression) to fatigue severity at 6 month follow-up. Sensitivity analysis, sensitivity dataset #2  Table S7. Baseline predictors of fatigue severity at six months follow-up. Final multiple linear regression model. Sensitivity analysis, sensitivity dataset #1  **Table S8**. Baseline predictors of fatigue severity at six months follow-up. Final multiple linear regression model. Sensitivity analysis, sensitivity dataset #2  **Table S9.** Baseline predictors and their adjusted associations (multiple linear regression) to fatigue severity at 6 month follow-up.  **Figure S1** Correlation heatmap of independent predictor variables with p<0.2 in bivariate analyses. | |  |
|  |  |  |
|  |  |  |
| **3. References – supplementary** | |  |

**1. Materials and methods – supplementary**

Further details of the study’s design, recruitment, data collection including sampling of biological specimens and laboratory assays, are given elsewhere^1^.

***Sampling and biorepository procedures*** *– please note that this section has previously been published in the supplemental documents of a previous paper*^1^

The primary biological specimens obtained were blood, hair, urine, and stool. Blood samples were obtained from antecubital venous puncture. If requested by the participants, local anaesthetic ointment (EMLA®) was applied for at least 60 minutes, but removed 15 minutes prior to sampling. A hair sample was collected from the parietal/occipital region of the scalp, where a bundle of hair with approximately the same diameter as a pencil was cut as close to the scalp as possible. Urine and stool samples were collected by the participants themselves, in the morning on the day of investigation.

Blood samples for routine analysis were immediately delivered to the accredited laboratory at Akershus University Hospital, Norway. Blood samples for storage at the biorepository underwent further preparations in order to obtain aliquots of plasma, serum, whole blood, RNA and viable Peripheral Blood Mononuclear Cells (PBMC). Thereafter, blood derived material not subjected to further analyses as well as the hair, urine and stool samples were transferred to a biorepository adjacent to the study centre (EpiGen laboratories, Akershus University Hospital, Norway), and stored at –80 ^o^C or –150 ^o^C, as appropriate. Results from analyses of whole blood, RNA, PBMC, hair, urine and stool are not reported in the present paper.

*Cytokines, growth factors and complement activation markers*

EDTA whole blood samples were placed on ice-water for 5-60 minutes. Thereafter, plasma was separated by centrifugation (2200 g, 10 min.) and frozen at –80 °C until assayed. Plasma samples were analyzed using a multiplex cytokine assay (Bio-Plex Human Cytokine 27-Plex Panel; Bio-Rad Laboratories Inc., Hercules, CA, USA) containing the following cytokines: IL-1β, IL-1 receptor antagonist (IL1-ra), IL-2, IL-4, IL-5, IL-6, IL-7, IL-8, IL-9, IL-10, IL-12, IL-13, IL-15, IL-17A, eotaxin, basic fibroblast growth factor (bFGF), granulocyte-colony stimulating factor (G-CSF), granulocyte macrophage colony stimulating factor (GM-CSF), interferon (IFN)-γ, interferon-inducible protein (IP-10), monocyte chemotactic protein (MCP-1), macrophage inflammatory protein (MIP)-1α, MIP-1β, platelet derived growth factor-BB (PDGF-BB), regulated upon activation T cell expressed and secreted (RANTES), Tumor Necrosis Factor (TNF), and vascular endothelial growth factor (VEGF). The samples were analyzed on a Multiplex Analyser (Bio-Rad Laboratories) according to instructions from the manufacturer. Due to a large amount of missing data (>50%), eight cytokines (IL-1ra, IL-5, IL-6, IL-10, IL-15, G-CSF, PDGF-BB and VEGF) were excluded from further analyses.

Plasma levels of growth/differentiation factor (GDF)-15 and C-reactive protein (CRP) were measured in duplicate by enzyme immunoassays (EIA) using commercially available antibodies (R&D Systems, Minneapolis, MN, USA) in a 384-format using a combination of a SELMA (Jena, Germany) pipetting robot and a BioTek (Winooski, VT) dispenser/washer. Absorption was read at 450 nm with wavelength correction set to 540 nm using an ELISA plate reader (BioTek, Winooski, VT).

The complement activation products C3bc and the terminal complement complex (TCC) sC5b-9 were quantified in plasma using enzyme-linked immunosorbent assays (ELISAs) based on monoclonal antibodies designed against neoepitopes of the products, not reacting with the native component.^2^ The units of these two well-established in-house assays are given according to an international standard defined as complement activation units (CAU) per milliliter with blood donors to define upper reference values of the normal population.

*SARS-CoV-2-antibodies*

Serum samples were tested with the Elecsys® Anti-SARS-CoV-2 immunoassay (Roche Diagnostics, Cobas e801, Mannheim, Germany) for IgG/IgM against the SARS-CoV-2 nucleocapsid antigen. The specificity and the sensitivity of the test are estimated by the manufacturer as 99.8% and 99.5%, respectively. In addition, antibodies to full-length spike protein (Spike-FL) and the receptor-binding domain (RBD) were measured using a multiplexed bead-based assay described in detail earlier.^3^ Briefly, sera were diluted 1:100 and incubated for 30 min with polymer beads with fluorescent bar codes coupled to Spike-FL or RBD. The beads were next washed, and aliquots were labelled with R-Phycoerythrin-conjugated anti-human IgG Fc (Jackson ImmunoResearch, West Grove, PA) and analyzed by flow cytometry (Attune Next, Thermo Fisher Scientific, Waltham, MA). The median fluorescence intensity (MFI) of beads coupled with viral antigens was divided by the MFI measured for beads with no antigen. Effects of sera on ACE2-binding to RBD were measured as a proxy for neutralizing antibodies. The beads were incubated with sera as described above, but labelled with digoxigenin-conjugated ACE2 and R-Phycoerythrin-conjugated anti-digoxigenin (Jackson ImmunoResearch, West Grove, PA). Signals measured in sera with no detectable anti-RBD were used as reference for no inhibitory effect.

*Epstein-Barr virus antibodies*

Specific antibody responses were assessed in serum samples using EBV VCA IgM and IgG (LIAISON®, DiaSorin, Saluggia, Italy) and EBV EBNA IgG (LIAISON®, DiaSorin, Saluggia, Italy). A rapid chromatographic immunoassay for the qualitative detection of Infectious Mononucleosis heterophile antibodies, Clearview® IM II (Abbott Laboratories, USA), was performed on serum samples with inconclusive result from the three specific tests. The specificity and the sensitivity of EBV-VCA IgM are estimated by the manufacturer as 99.2 % and 97.8 %, respectively; for EBV-VCA IgG 95.8 % and 98.5 %, respectively; and for EBV-EBNA IgG 97.6 % and 98.8 %, respectively. The manufacturer states >99 % negative and positive agreement between Clearview® IM II and slide agglutination.

*Brain injury markers*

Blood for neurofilament light chain (NfL) and glial fibrillary acidic protein (GFAp) measurements in serum was collected in 3,5 mL Vacuette R (Greiner Bio-One GmbH, Kremsmünster, Austria) with gel, allowed to clot for at least 30 minutes, processed within 2 hours by centrifugation (2200 g, 10 min) and aliquots stored immediately at − 80 °C until analysis. Serum GFAp and NfL measurements were performed at the Clinical Neurochemistry Laboratory, Sahlgrenska University Hospital, Sweden, by board-certified laboratory technicians blinded to clinical data using commercially available Single molecule array (Simoa) assays on an HD-X Analyzer (Human Neuro 2-Plex B assay), as described by the manufacturer (Quanterix, Billerica, MA). Calibrators were run in duplicates, while samples were diluted 4-fold and run in singlicates. Two quality control (QC) samples with different levels were run in duplicates in the beginning and the end of each run. Repeatability and intermediate precision were both 8.7% for the QC sample with an NfL concentration of 8.4 pg/mL and 5.9% for the 79.6 pg/mL sample. For GFAP, repeatability was 6.5% and intermediate precision 7.3% for the QC sample at 102 pg/mL, and repeatability was 5.8% and intermediate precision 6.7% for the QC sample at 388 pg/mL.

*Routine blood analyses*

Routine blood analyses were assayed at the accredited laboratory at Akershus University Hospital, Norway, and included the following markers: haemoglobin; leukocytes with differential count; platelets; CRP; ferritin; alanine transaminase (ALT); gamma-glutamyltransferase (GGT); lactate dehydrogenase (LDH); albumin; N-terminal prohormone of Brain Natriuretic Peptide (NT-proBNP); troponin T; creatine kinase (CK); glucose; glycated haemoglobin (HbA_1C_); bilirubin; D-dimer; international normalized ratio (INR); urea; creatinine; natrium; potassium; calcium; vitamin B_12_; folic acid; thyroid-stimulating hormone (TSH); thyroxine; cortisol; IgG (total); IgM (total); IgA (total); blood gases (venous sample); SARS-CoV-2 total antibody titer (IgM+IgG).

**Statistical analyses – main data set and data sets for sensitivity analyses**

*The per protocol data set*

The per protocol data set was defined as all individuals completing the investigational program at baseline and six months follow up, except:

- SARS-CoV-2-negative individuals at baseline with reported SARS-CoV-2 infection in the observational period or anti-SARS-CoV-2 antibodies (any type for unvaccinated, anti-nucleocapsid for vaccinated) detected at six months follow-up.
- SARS-CoV-2-positive individuals at baseline with reported novel SARS-CoV-2 infection in the observational period, or increased anti-nucleocapsid antibody-titer at six months as compared to baseline.

These individuals were thought to violate a fundamental premise of the study (one acute SARS-CoV-2 infection in the SARS-CoV-2-positive group, no acute SARS-CoV-2 infection in the SARS-CoV-2-negative group) and were therefore excluded from all further analyses. In the per-protocol data set, laboratory values below lower detection limit (LDL) were replaced with a random value in the interval between zero and LDL for each specific analysis. Otherwise, no missing data were imputed.

*Data set for sensitivity analyses #1 – multiple imputation of missing values*

For sensitivity analyses purposes, multiple imputed data sets were constructed based upon the per protocol data set. All missing values were imputed using predictive mean matching and the MICE package.^4^

*Data set for sensitivity analyses #2 – removal of potential bias*

A second dataset was created to remove potential source of bias, i.e. other conditions/states that may give rise to fatigue. The following potential sources of bias were identified, and individuals with the corresponding properties were removed:

1. The impact of immunisation against SARS-CoV-2 for development of long COVID is unclear. Some data suggests a protective effect,^5^ whereas others have speculated that vaccination may actually trigger PIFS in vulnerable individuals, as has been reported after immunization against other microorganisms.^6^ Also, common side effects in the days after vaccination (chills, malaise, etc.) may mimic the symptoms of long COVID. Hence individuals that received vaccination prior to inclusion or less than five days prior to the six-month follow-up appointment were excluded.
2. Epstein-Barr Virus (EBV) infection is the only endemic infection in Norway that has a documented association to PIFS development.^60^ Thus, acute EBV-infection among the participants of the present study may bias the results. Individuals with EBV serology results suggesting acute EBV infection at inclusion or during the six months observational period, or for which an early infection at the six-month follow-up could not be ruled out, were excluded.
3. Fatigue can be regarded as one of the core symptoms of major depressive disorder^7^ and thus pre-existing depression represents a source of bias in the study. Individuals with a score > 15 on the depression subscale of the hospital and anxiety depression score at baseline were excluded from the dataset.
4. Fatigue can be a symptom or sequelae of a number of diseases.^8^ To minimise bias from fatigue due to comorbidities, we identified individuals with pre-existing complex chronic conditions^9^ or pain/fatigue-related comorbidities (migraine / chronic headache, irritable bowel syndrome, endometriosis, chronic fatigue syndrome). Medical records were reviewed, and those with evidence of pre-existing fatigue, or with missing records, were excluded.

**2. Results – supplementary**

| Table S1. Analyses of missing data. Characteristics of independent variables and their association to complete cases at six months follow-up. Logistic regression. | | | | | | | | |
| --- | --- | --- | --- | --- | --- | --- | --- | --- |
|  |  | | **Baseline characteristics** | | **Odds ratio of being a complete case at six months** | | |  |
|  | *Cases with available data for variable, N (%))* | *All cases with available data for variable (n=N)* | | *Complete cases only (n=307)* | *Odds ratio (CI)* | *p-value* |  | |
| SARS-CoV-2 status |  |  | |  |  |  |  | |
| SARS-CoV-2-positive at baseline – no. (%) | 467 | 382 (81.8) | | 247 (80.2) | 0.72 (0.423, 1.19) | 0.213 |  | |
| Background and constitutional factors |  |  | |  |  |  |  | |
| Female sex – no. (%) | 467 | 284 (60.8) | | 197 (64) | 1.47 (0.995, 2.17) | 0.053 |  | |
| Age, years – mean (CI) | 467 | 17.9 (17.6, 18.3) | | 18.2 (17.8, 18.6) | 1.06 (1, 1.12) | 0.039 |  | |
| BMI, z-score^a^  – mean (CI) | 466 | 0.444 (0.339, 0.549) | | 0.547 (0.429, 0.665) | 1.26 (1.07, 1.5) | 0.007 |  | |
| Ethnicity non-European – no. (%) | 467 | 90 (19.3) | | 47 (15.3) | 0.486 (0.304, 0.777) | 0.002 |  | |
| Asthma – no.(%) | 452 | 30 (6.64) | | 17 (5.52) | 0.589 (0.279, 1.27) | 0.167 |  | |
| Any comorbidity – no. (%) | 453 | 107 (23.6) | | 71 (23.1) | 0.907 (0.575, 1.45) | 0.678 |  | |
| Observational period characteristics |  |  | |  |  |  |  | |
| Time span between baseline and follow-up, days – median (range) | 467 | 193 (188, 203) | | 194 (188, 206) | 1.01 (0.999, 1.02) | 0.095 |  | |
| Immunisation against SARS-CoV-2^b^ – no. (%) | 467 | 7 (1.5) | | 4 (1.3) | 0.684 (0.149, 3.51) | 0.622 |  | |
| Organ function tests/biomarkers |  |  | |  |  |  |  | |
| FVC, % of predicted^c^ – mean (CI) | 400 | 99.7 (98.7, 101) | | 99.1 (97.9, 100) | 0.974 (0.952, 0.996) | 0.022 |  | |
| SpO_2_, % – mean (CI) | 465 | 98.7 (98.6, 98.8) | | 98.6 (98.5, 98.8) | 0.995 (0.835, 1.18) | 0.951 |  | |
| Systolic blood pressure, mmHg – mean (CI) | 464 | 117 (116, 118) | | 118 (117, 119) | 1.02 (1.01, 1.04) | 0.009 |  | |
| Diastolic blood pressure | 464 | 72.7 (72.1, 73.4) | | 73.1 (72.4, 73.9) | 1.02 (0.994, 1.05) | 0.128 |  | |
| NT-pBNP^*^, ng/L – median (CI) | 439 | 35 (21.5, 57) | | 34 (21, 54) | 0.996 (0.99, 1) | 0.190 |  | |
| Troponin T, ng/L – median (CI) | 447 | 4 (2.26, 5) | | 4 (2.22, 6) | 0.961 (0.894, 1.03) | 0.272 |  | |
| NfL^*^, pg/mL – mean (CI) | 461 | 4.63 (4.3, 4.96) | | 4.69 (4.24, 5.13) | 1.02 (0.961, 1.1) | 0.627 |  | |
| GFAp^*^, pg/mL – mean (CI) | 461 | 67.4 (62.9, 72) | | 66.7 (60.7, 72.8) | 0.999 (0.995, 1) | 0.663 |  | |
| D-dimer^*^, mg/L – median (CI) | 456 | 0.179 (0.0805, 0.268) | | 0.177 (0.0811, 0.261) | 1.07 (0.345, 3.72) | 0.909 |  | |
| Ferritin^*^, µg/L – median (CI) | 437 | 66 (42, 101) | | 67 (42, 106) | 1 (1, 1.01) | 0.024 |  | |
| Vitamin B_12_^†^, pmol/L – mean (CI) | 443 | 440 (424, 455) | | 431 (413, 449) | 0.999 (0.998, 1) | 0.097 |  | |
| Vitamin D^*^, - median (CI) | 456 | 52.5 (39, 66.2) | | 53.5 (41, 66) | 1 (0.993, 1.01) | 0.681 |  | |
| HbA1c^*^, mean (CI) | 443 | 33.6 (33.3, 33.9) | | 33.5 (33.1, 33.8) | 0.969 (0.913, 1.03) | 0.293 |  | |
| Immunological markers |  |  | |  |  |  |  | |
| Blood Leukocyte count^*^, 10^9^ cells/L - mean (CI) | 427 | 5.87 (5.73, 6.01) | | 5.87 (5.71, 6.04) | 1.01 (0.877, 1.16) | 0.922 |  | |
| Blood Lymphocyte count, 10^9^ cells/L - mean (CI) | 437 | 2.12 (2.06, 2.17) | | 2.12 (2.05, 2.18) | 0.985 (0.693, 1.41) | 0.936 |  | |
| Blood Monocyte count^*^, 10^9^ cells/L - mean (CI) | 438 | 0.448 (0.434, 0.462) | | 0.446 (0.429, 0.464) | 0.828 (0.217, 3.26) | 0.784 |  | |
| Blood Neutrophil count^*^, 10^9^ cells/L - mean (CI) | 437 | 3.14 (3.04, 3.25) | | 3.13 (3.01, 3.26) | 0.973 (0.815, 1.17) | 0.761 |  | |
| Neutrophil-to-Lymphocyte ratio^*^ – mean (CI) | 437 | 1.45 (1.1, 1.86) | | 1.46 (1.07, 1.86) | 0.982 (0.723, 1.34) | 0.906 |  | |
| Systemic immune-inflammation index ^d*^- median (CI) | 428 | 375 (267, 502) | | 368 (263, 494) | 1 (0.999, 1) | 0.486 |  | |
| Plasma total IgA, g/L - mean (CI) | 451 | 1.68 (1.61, 1.75) | | 1.71 (1.63, 1.8) | 1.17 (0.899, 1.53) | 0.249 |  | |
| Plasma total IgM^†^, g/L - mean (CI) | 452 | 1.24 (1.19, 1.29) | | 1.24 (1.18, 1.29) | 0.976 (0.673, 1.43) | 0.900 |  | |
| Plasma total IgG^†^, g/L - mean (CI) | 450 | 11 (10.8, 11.2) | | 10.9 (10.7, 11.1) | 0.932 (0.85, 1.02) | 0.136 |  | |
| hsCRP^*^, mg/L – median (CI) | 451 | 0.887 (0.388, 2.59) | | 0.94 (0.392, 2.61) | 1.03 (0.961, 1.1) | 0.455 |  | |
| Plasma IL-1β^*^, pg/mL – median (CI) | 451 | 0.47 (0.008, 0.98) | | 0.47 (0.00875, 0.98) | 1.01 (0.801, 1.28) | 0.961 |  | |
| Plasma IL-2^‡,^ pg/mL - median (CI) | 451 | 0.69 (0.0217, 1.66) | | 0.66 (0.022, 1.66) | 1.01 (0.883, 1.15) | 0.937 |  | |
| Plasma IL-4, pg/mL - median (CI) | 451 | 1.33 (0.9, 1.82) | | 1.33 (0.9, 1.78) | 0.961 (0.75, 1.24) | 0.754 |  | |
| Plasma IL-7^†^, pg/mL - median (CI) | 451 | 11.5 (2.42, 17.2) | | 12.2 (2.42, 17.2) | 1.02 (0.997, 1.04) | 0.124 |  | |
| Plasma IL-8^‡^, pg/mL - median (CI) | 451 | 0.55 (0.0922, 1.96) | | 0.565 (0.0924, 1.91) | 1.02 (0.936, 1.13) | 0.702 |  | |
| Plasma IL-9^†^, pg/mL - median (CI) | 451 | 68.9 (27.2, 157) | | 71.2 (26.6, 160) | 1 (0.999, 1) | 0.734 |  | |
| Plasma IL-12^†^, pg/mL - median (CI) | 451 | 1.38 (0.151, 4.84) | | 1.38 (0.177, 4.84) | 0.993 (0.951, 1.04) | 0.735 |  | |
| Plasma IL-13^*^, pg/mL - median (CI) | 451 | 0.27 (0.019, 0.73) | | 0.27 (0.0197, 0.86) | 1.19 (0.98, 1.52) | 0.124 |  | |
| Plasma IL-17A^*^, pg/mL - median (CI) | 451 | 1.62 (0.3, 2.98) | | 1.62 (0.236, 2.87) | 0.949 (0.857, 1.05) | 0.322 |  | |
| Plasma TNF, pg/mL - median (CI) | 450 | 6.73 (3.36, 11.5) | | 6.73 (3.35, 11.5) | 0.992 (0.966, 1.02) | 0.546 |  | |
| Plasma IFN-γ^*^, pg/mL - median (CI) | 451 | 1.14 (0.4, 1.97) | | 1.14 (0.4, 2.06) | 1.02 (0.97, 1.11) | 0.455 |  | |
| MCP-1/CCL2, pg/mL – mean (CI) | 451 | 12.2 (8.95, 16) | | 12.2 (8.95, 16.2) | 1 (0.973, 1.04) | 0.787 |  | |
| IP-10^*^, pg/mL – mean (CI) | 451 | 157 (149, 165) | | 156 (148, 164) | 1 (0.997, 1) | 0.698 |  | |
| Plasma Eotaxin-1/CCL11^*^, pg/mL - median (CI) | 451 | 14.1 (11.2, 18.5) | | 14.1 (10.9, 18.5) | 0.993 (0.965, 1.02) | 0.646 |  | |
| Plasma MIP-1α^†^, pg/mL - median (CI) | 451 | 0.77 (0.56, 1) | | 0.77 (0.56, 1) | 1.3 (0.779, 2.19) | 0.323 |  | |
| Plasma MIP-1β^†^, pg/mL - median (CI) | 451 | 24.9 (12.4, 49.5) | | 24.7 (12.3, 50.6) | 1 (0.997, 1.01) | 0.643 |  | |
| RANTES/CCL5^*^, pg/mL – median (CI) | 451 | 264 (122, 523) | | 265 (120, 523) | 1 (1, 1) | 0.442 |  | |
| Plasma GM-CSF^*^, pg/mL - median (CI) | 451 | 0.11 (0.0162, 0.46) | | 0.06 (0.0154, 0.46) | 1.05 (0.935, 1.21) | 0.462 |  | |
| Plasma bFGF^*^, pg/mL - median (CI) | 451 | 2.4 (1.32, 7.31) | | 2.3 (1.32, 7.31) | 1 (0.972, 1.03) | 0.995 |  | |
| GDF15^*^, ng/mL – mean (CI) | 451 | 0.405 (0.389, 0.421) | | 0.4 (0.382, 0.418) | 0.594 (0.193, 1.87) | 0.360 |  | |
| TCC/C5b-9^*^, CAU/mL – median (CI) | 451 | 0.16 (0.03, 0.27) | | 0.16 (0.04, 0.27) | 1.08 (0.902, 1.55) | 0.528 |  | |
| Plasma C3bc^†^, ng/mL - median (CI) | 450 | 3.64 (2.6, 4.79) | | 3.68 (2.6, 4.78) | 0.988 (0.877, 1.12) | 0.840 |  | |
| EBV prior infection^e^ – no. (%) | 461 | 328 (71.1) | | 215 (69.8) | 0.818 (0.526, 1.26) | 0.366 |  | |
| SARS-CoV-2-Anti-RBD^*^, BAU/mL – median (CI) | 461 | 130 (1, 1430) | | 212 (1, 1560) | 1 (1, 1) | 0.652 |  | |
| Autonomic markers |  |  | |  |  |  |  | |
| LF-RRI^*^, ms^2^ – median (CI) | 463 | 642 (338, 1180) | | 709 (370, 1280) | 1 (1, 1) | 0.028 |  | |
| HF-RRI^*^, ms^2^ – median (CI) | 463 | 810 (383, 1740) | | 836 (379, 1770) | 1 (1, 1) | 0.442 |  | |
| Cognitive function tests |  |  | |  |  |  |  | |
| Digit span^f^, total score – median (CI) | 464 | 15 (12, 17) | | 15 (13, 18) | 1.07 (1.01, 1.13) | 0.027 |  | |
| Immediate recall^g^, score 0 to 36 – median (CI) | 464 | 25 (22, 28) | | 25 (22, 28) | 1.05 (1, 1.1) | 0.036 |  | |
| Delayed recall^g^, score 0 to 12 – median (CI) | 464 | 9 (7, 10) | | 9 (7, 10) | 1.12 (1.01, 1.23) | 0.025 |  | |
| Recognition index^h^, score 0 to 12 – median (CI) | 463 | 12 (11, 12) | | 12 (11, 12) | 1.12 (0.916, 1.36) | 0.264 |  | |
| Clinical symptoms |  |  | |  |  |  |  | |
| Fatigue^i§^, score 0 to 33 – mean (CI) | 451 | 15.6 (15.1, 16.1) | | 15.5 (14.9, 16.1) | 0.989 (0.955, 1.02) | 0.537 |  | |
| Post-exertional malaise^j^, score 0 to 100 – median (CI) | 451 | 20 (5, 45) | | 15 (5, 40) | 0.999 (0.992, 1.01) | 0.894 |  | |
| Sleep problems^k^, score 1 to 6 – mean (CI) | 451 | 4.01 (3.9, 4.11) | | 4 (3.88, 4.13) | 0.989 (0.829, 1.18) | 0.898 |  | |
| Pain^l^, score 1 to 10 – median (CI) | 451 | 2.25 (1.5, 3.25) | | 2.25 (1.5, 3.25) | 0.978 (0.839, 1.14) | 0.781 |  | |
| Cognitive symptoms^m^, score 3 to 15 – median (CI) | 451 | 6 (4, 9) | | 6 (4, 8) | 0.949 (0.892, 1.01) | 0.097 |  | |
| Respiratory symptoms^n^, score 2 to 10 – median (CI) | 451 | 4 (3, 6) | | 4 (3, 5) | 0.942 (0.857, 1.04) | 0.220 |  | |
| Autonomic symptoms^o^, score 2 to 10 - median (CI) | 451 | 5 (4, 8) | | 5 (4, 8) | 1.01 (0.94, 1.08) | 0.828 |  | |
| Symptoms of anxiety^p^, score 0 to 21 – median (CI) | 451 | 6 (3, 9) | | 6 (3, 9) | 1.02 (0.973, 1.07) | 0.400 |  | |
| Symptoms of depression^p^, score 0 to 21 – median (CI) | 451 | 3 (1, 6) | | 3 (1, 6) | 0.946 (0.899, 0.996) | 0.035 |  | |
| Negative emotions^q^, score 5 to 25 – median (CI) | 451 | 10 (6, 15) | | 10 (6, 15) | 1.01 (0.97, 1.05) | 0.667 |  | |
| Psychological traits | 451 |  | |  |  |  |  | |
| Neuroticism^s^, score 0 to 24 – median (CI) | 451 | 6 (2, 12) | | 6 (2, 12) | 0.986 (0.955, 1.02) | 0.379 |  | |
| Emotional awareness^t^, score 7 to 35 – median (CI) | 451 | 13 (9, 20) | | 13 (9, 20) | 0.991 (0.961, 1.02) | 0.539 |  | |
| Worrying tendencies^u§^, score 16 to 80 – mean (CI) | 451 | 45.5 (44.2, 46.8) | | 45.9 (44.3, 47.4) | 1.01 (0.991, 1.02) | 0.487 |  | |
| Body vigilance^v^, score 0 to 40 – mean (CI) | 451 | 12 (11.3, 12.7) | | 12 (11.2, 12.9) | 1 (0.976, 1.03) | 0.836 |  | |
| Social/behavioural markers |  |  | |  |  |  |  | |
| Average level of physical activity prior to acute infection^x^, score 1 to 10 – mean (CI) | 451 | 6.37 (6.17, 6.58) | | 6.6 (6.37, 6.83) | 1.16 (1.06, 1.27) | 0.002 |  | |
| Socioeconomic level ISEI-08^y^, score 10 to 90 – median (CI) | 423 | 63.3 (39, 75.1) | | 66 (43.3, 76.5) | 1.01 (1, 1.03) | 0.006 |  | |
| Family member with chronic disease^z^ – no. (%) | 451 | 153 (33.9) | | 103 (33.4) | 0.935 (0.617, 1.42) | 0.751 |  | |
| Loneliness^aa^, score 20-80 – mean (CI) | 451 | 38 (37, 39) | | 37.4 (36.2, 38.6) | 0.985 (0.967, 1) | 0.100 |  | |
| Negative life events last 12 months^ab^, impact score – median (CI) | 451 | 2 (0, 5) | | 2 (0, 6) | 1.02 (0.977, 1.08) | 0.337 |  | |
| Negative life events prior to last 12 months^ab^, impact score – median (CI) | 451 | 0 (0, 3) | | 0 (0, 3) | 1.05 (0.956, 1.15) | 0.336 |  | |
| *Dependent variable* |  |  | |  |  |  |  | |
| *Fatigue^i^, score 0 to 33 – mean (CI) at six months* | *467* | *382 (81.8)* | | *247 (80.2)* | *0.72 (0.423, 1.19)* | *0.213* |  | |
| CI=95% Confidence interval; NA=Not applicable; SARS-CoV-2= Severe acute respiratory syndrome coronavirus 2; BMI=Body mass index; FVC=Forced vital capacity; SpO_2_=Peripheral oxygen saturation; NT-pBNP=N-terminal pro-brain natriuretic peptide; NfL=Neurofilament light chain; GFAp=Glial fibrillary acidic protein; hsCRP=high-sensitive assay of C-reactive protein; GDF-15=Growth/differentiation factor 15; IL=Interleukin; TCC=Terminal complement complex; CAU=Complement arbitrary units; RANTES=Regulated on activation, normal T-cell expressed and secreted; MCP=Monocyte chemotactic protein; IP=Interferon gamma-induced protein; RBD= Receptor binding domain; BAU=Binding antibody units; LF-RRI=Low frequency power of heart rate variability; HF-RRI=High-frequency power of heart rate variability; ISEI-08=International Socioeconomic Index 2008. ^a^ As measured by the Chalder Fatigue Questionnaire, score 0-33, higher scores imply more fatigue. Log_e_(x+1) transformation was used for regression analyses. ^a^Standardised score calculated according to World Health Organisation 2006 Child Growth Standards for ages 12-19; for participants above this age, reference values for 19-year-olds were used. ^b^One or more doses of immunisation against SARS-CoV-2. ^c^The Global Lung Function Initiative 2012 reference values were used to calculate predicted values. ^d^Defined as (NxP)/L, where N, P and L represent neutrophil, platelet and lymphocyte counts respectively. ^e^Positive IgG antibodies (VCA and/or EBNA) and negative heterophile antibodies at baseline and six months. ^f^From the Wechsler Intelligence Scales for Children revised; higher score implies better short-term memory. ^g^From the Hopkins Verbal Learning Test revised (HVLT-R); higher scores imply better immediate and delayed recall of words, respectively. ^h^From the HVLT-R; higher score implies better recognition of words. ^i^From the Chalder Fatigue Questionnaire; higher score implies more fatigue. ^j^From the DePaul Symptom Questionnaire; higher score implies more post-exertional malaise. ^k^From the Karolinska Sleep Questionnaire; higher score implies better sleep. ^l^From the Brief Pain Inventory, higher score implies more pain. ^m^Self-developed, aggregated score for problems with ‘memory’, ‘concentration’, and ‘decision making’; higher score implies more symptoms. ^n^Self-developed, aggregated score for symptoms ‘cough’ and ‘dyspnoea’; higher score implies more symptoms. ^o^Self-developed, aggregated score for symptoms ‘dizziness’, ‘cold and pale hands’, ‘feeling alternately warm and cold’; higher score implies more symptoms. ^p^From the anxiety and depression subscales, respectively, of the Hospital Anxiety and Depression Scale; higher scores imply more symptoms. ^q^From the Positive and Negative Affect Schedule; higher score implies more negative emotions. ^r^The main component extracted by Principal Component Analysis of the 10 clinical symptoms variables, labelled ‘symptom severity’. ^s^From the NEO-Five-Factor-Inventory-30; higher scores implies more neuroticism. ^t^From the Toronto Alexithymia Scale; higher score implies more difficulty identifying feelings. ^u^From the Penn State Worry Questionnaire; higher score implies more worrying. ^v^From the Body Vigilance Scale; higher score implies being more attentive to bodily sensations. ^w^The main component extracted by Principal Component Analysis of the four psychological traits variables, labelled ‘emotional maladjustment’. ^x^Self-developed; higher score implies more physical activity. ^y^The ISEI-08 score of the parent with the highest score; higher score implies higher socioeconomic status. ^z^Having a sibling or parent affected by chronic disease. ^aa^From the University of California, Los Angeles, Loneliness Scale; higher score implies more loneliness. ^ab^From the Life Event Checklist; higher score implies more negative impact of past life events. | | | | | | | | |

| Table S2. Results of final factor analyses (Principal Component Analysis) of ten ‘clinical symptoms’ variables and four ‘psychological traits’ variables, respectively. Per protocol data. | | |
| --- | --- | --- |
|  | **Principal component from clinical symptoms variables: ‘Symptom severity’** | **Principal component from psychological traits variables: ‘Emotional maladjustment’** |
| Total variance explained (%) | 52.7 | 66.4 |
| Bartlett’s test of sphericity (p-value) | <0.001 | <0.001 |
| Kaiser-Meyer-Olkin measure of sampling adequacy | 0.91 | 0.74 |
| Loading variables |  |  |
| Fatigue^a^ (factor loading) | 0.832 |  |
| Post-exertional malaise^b^ (factor loading) | 0.818 |  |
| Sleep problems^c^ (factor loading) | -0.817 |  |
| Pain^d^ (factor loading) | 0.613 |  |
| Cognitive symptoms^e^ (factor loading) | 0.770 |  |
| Respiratory symptoms^f^ (factor loading) | 0.538 |  |
| Autonomic symptoms^g^ (factor loading) | 0.762 |  |
| Symptoms of anxiety^h^ (factor loading) | 0.749 |  |
| Symptoms of depression^h^ (factor loading) | 0.679 |  |
| Negative emotions^i^ (factor loading) | 0.633 |  |
| Neuroticism^j^ (factor loading) |  | 0.903 |
| Emotional awareness^k^ (factor loading) |  | 0.833 |
| Worrying tendencies^l^ (factor loading) |  | 0.861 |
| Body vigilance^m^ (factor loading) |  | 0.637 |
| ^a^From the Chalder Fatigue Questionnaire; higher score implies more fatigue. ^b^From the DePaul Symptom Questionnaire; higher score implies more post-exertional malaise. ^c^From the Karolinska Sleep Questionnaire; higher score implies better sleep. ^d^From the Brief Pain Inventory, higher score implies more pain. ^e^Self-developed, aggregated score for problems with ‘memory’, ‘concentration’, and ‘decision making’; higher score implies more symptoms. ^f^Self-developed, aggregated score for symptoms ‘cough’ and ‘dyspnoea’; higher score implies more symptoms. ^g^Self-developed, aggregated score for symptoms ‘dizziness’, ‘cold and pale hands’, ‘feeling alternately warm and cold’; higher score implies more symptoms. ^h^From the anxiety and depression subscales, respectively, of the Hospital Anxiety and Depression Scale; higher scores imply more symptoms. ^i^From the Positive and Negative Affect Schedule; higher score implies more negative emotions. ^j^From the NEO-Five-Factor-Inventory-30; higher scores implies more neuroticism. ^k^From the Toronto Alexithymia Scale; higher score implies more difficulty identifying feelings. ^l^From the Penn State Worry Questionnaire; higher score implies more worrying. ^m^From the Body Vigilance Scale; higher score implies being more attentive to bodily sensations. | | |

| Table S3. Baseline independent variables associated with fatigue severity at six months follow-up. Overview of results from final multiple linear regression models in 45 imputed sets^a^. Sensitivity analysis using predicted mean matching to impute all missing values in the original dataset. N = 467 | | | | | | | | | | | | | | | | | | | | | | | | | | | | | | | | | | | | | | | | | | | | | |
| --- | --- | --- | --- | --- | --- | --- | --- | --- | --- | --- | --- | --- | --- | --- | --- | --- | --- | --- | --- | --- | --- | --- | --- | --- | --- | --- | --- | --- | --- | --- | --- | --- | --- | --- | --- | --- | --- | --- | --- | --- | --- | --- | --- | --- | --- |
|  | ***Imputed set number*** | | | | | | | | | | | | | | | | | | | | | | | | | | | | | | | | | | | | | | | | | | | | |
| ***Variable:*** | 1 | 2 | 3 | 4 | 5 | 6 | 7 | 8 | 9 | 10 | 11 | 12 | 13 | 14 | 15 | 16 | 17 | 18 | 19 | 20 | 21 | 22 | 23 | 24 | 25 | 26 | 27 | 28 | 29 | 30 | 31 | 32 | 33 | 34 | 35 | 36 | 37 | 38 | 39 | 40 | 41 | 42 | 43 | 44 | 45 |
| Ferritin | 1 |  |  |  |  |  |  |  |  |  |  |  |  |  |  |  |  |  |  |  |  |  |  |  |  |  |  |  |  |  |  |  |  |  |  |  |  |  |  |  |  |  |  |  |  |
| Plasma IL-4 | 1 | 1 |  | 1 | 1 | 1 | 1 | 1 | 1 | 1 | 1 | 1 | 1 | 1 | 1 | 1 |  | 1 | 1 | 1 | 1 | 1 | 1 | 1 | 1 | 1 | 1 | 1 | 1 | 1 | 1 | 1 | 1 | 1 | 1 | 1 | 1 | 1 | 1 | 1 | 1 | 1 | 1 | 1 | 1 |
| Plasma IL-7 | 1 | 1 | 1 | 1 | 1 | 1 | 1 | 1 | 1 | 1 |  | 1 | 1 | 1 | 1 | 1 | 1 | 1 | 1 | 1 | 1 | 1 | 1 | 1 | 1 | 1 | 1 | 1 | 1 |  | 1 |  | 1 | 1 | 1 | 1 |  | 1 | 1 | 1 | 1 | 1 | 1 | 1 | 1 |
| Plasma C3bc | 1 | 1 | 1 | 1 | 1 | 1 | 1 | 1 | 1 | 1 | 1 | 1 | 1 | 1 | 1 | 1 | 1 | 1 | 1 | 1 | 1 | 1 | 1 | 1 | 1 | 1 | 1 | 1 | 1 | 1 | 1 | 1 | 1 | 1 | 1 | 1 | 1 | 1 | 1 | 1 | 1 | 1 | 1 | 1 | 1 |
| Symptom severity | 1 | 1 | 1 | 1 | 1 | 1 | 1 | 1 | 1 | 1 | 1 | 1 | 1 | 1 | 1 | 1 | 1 | 1 | 1 | 1 | 1 | 1 | 1 | 1 | 1 | 1 | 1 | 1 | 1 | 1 | 1 | 1 | 1 | 1 | 1 | 1 | 1 | 1 | 1 | 1 | 1 | 1 | 1 | 1 | 1 |
| Loneliness |  |  |  |  |  |  |  |  |  |  | 1 |  |  |  |  |  |  |  |  |  |  |  |  |  |  |  |  |  |  |  |  |  |  |  |  |  |  |  |  |  |  |  | 1 |  | 1 |
| Negative life events last 12 months |  |  |  |  |  |  |  |  |  |  | 1 |  |  |  |  |  |  |  |  |  |  |  |  |  |  |  |  |  |  |  |  |  |  |  |  |  |  |  |  |  |  |  | 1 |  |  |
| Socioeconomic level ISEI-08 |  |  |  |  |  |  |  |  |  |  |  |  |  |  |  |  |  |  |  |  |  | 1 |  |  |  |  |  |  | 1 |  |  |  |  |  |  |  |  |  |  |  |  |  |  |  |  |
| ^a^Variables included in the final model of the respective sets are marked in grey, while variables not included are white. Control variables, and variables not included in any of the 45 models, have been omitted from the table. | | | | | | | | | | | | | | | | | | | | | | | | | | | | | | | | | | | | | | | | | | | | | |

| Table S4. Characteristics of potential baseline predictors and their univariate associations to fatigue severity^*^ at 6-month follow-up. Linear regression. Per protocol data | | | | | | |
| --- | --- | --- | --- | --- | --- | --- |
|  | **Baseline characteristics** | | **Univariate association to fatigue severity** | | | |
|  | *SARS-CoV-2 positive (n=382)* | *SARS-CoV-2 negative (n=85)* | *Coefficent (CI)* | *SE* | p-value | *Adjusted R^2^(%)* |
| SARS-CoV-2 status |  |  |  |  |  |  |
| SARS-CoV-2-positive at baseline – no. (%) | NA | NA | 0.057 ( -0.03 to 0.14 ) | 0.044 | 0.202 | 0.1 |
| Background and constitutional factors |  |  |  |  |  |  |
| Female sex – no. (%) | 230 (60.2) | 54 (63.5) | 0.23 ( 0.16 to 0.3 ) | 0.033 | <0.001 | 9.0 |
| Age, years – mean (CI) | 17.98 (17.61, 18.35) | 17.73 (17.04, 18.43) | 0.017 ( 0.0077 to 0.026 ) | 0.005 | <0.001 | 2.5 |
| BMI, z-score^a^  – mean (CI) | 0.44 (0.32, 0.55) | .48 (0.23, 0.72) | -0.0016 ( -0.031 to 0.028 ) | 0.015 | 0.914 | -0.2 |
| Ethnicity non-European – no. (%) | 88 (23.0) | 2 (2.4) | -0.034 ( -0.12 to 0.051 ) | 0.043 | 0.435 | -0.1 |
| Asthma – no.(%) |  |  | 0.1 ( -0.032 to 0.24 ) | 0.069 | 0.135 | 0.3 |
| Any comorbidity – no. (%) | 79 (21.4) | 28 (33.3) | 0.074 ( -0.0055 to 0.15 ) | 0.040 | 0.068 | 0.5 |
| Observational period characteristics |  |  |  |  |  |  |
| Time span between baseline and follow-up, days – median (range) | 193 (191, 195) | 193 (190, 196) | 0.00023 ( -0.0015 to 0.002 ) | 0.001 | 0.799 | -0.2 |
| Immunisation against SARS-CoV-2^b^ – no. (%) | 4 (1.0) | 3 (3.5) | 0.26 ( -0.013 to 0.54 ) | 0.140 | 0.062 | 0.5 |
| Organ function tests/biomarkers |  |  |  |  |  |  |
| FVC, % of predicted^c^ – mean (CI) | 99.4 (98.3, 100.6) | 100.8 (98.4, 103.2) | -0.0026 ( -0.006 to 8e-04 ) | 0.002 | 0.134 | 0.3 |
| SpO_2_, % – mean (CI) | 98.67 (98.56, 98.78) | 98.57 (98.30, 98.84) | 0.018 ( -0.012 to 0.048 ) | 0.015 | 0.244 | 0.1 |
| Systolic blood pressure, mmHg – mean (CI) | 117 (116, 118) | 118 (115, 120) | -0.002 ( -0.0051 to 0.001 ) | 0.002 | 0.191 | 0.2 |
| Diastolic blood pressure | 73 (72, 73) | 73 (72, 75) | -0.00021 ( -0.005 to 0.0045 ) | 0.002 | 0.931 | -0.2 |
| NT-pBNP^**^, ng/L – median (CI) | 34 (30, 38) | 35 (26, 44) | 0.042 ( -0.0042 to 0.088 ) | 0.023 | 0.075 | 0.5 |
| Troponin T, ng/L – median (CI) | 4.00 (4.00, 4.00) | 2.89 (2.21, 4.00) | -0.087 ( -0.17 to -0.00082 ) | 0.044 | 0.048 | 0.7 |
| NfL^**^, pg/mL – mean (CI) | 4.73 (4.33, 5.12) | 4.20 (3.86, 4.54) | -0.029 ( -0.11 to 0.056 ) | 0.043 | 0.506 | -0.1 |
| GFAp^**^, pg/mL – mean (CI) | 70.02 (64.56, 75.48) | 56.02 (51.09, 60.95) | -0.031 ( -0.11 to 0.043 ) | 0.038 | 0.407 | -0.1 |
| D-dimer^**^, mg/L – median (CI) | 0.17 (0.15, 0.19) | 0.19 (0.17, 0.21) | -0.011 ( -0.042 to 0.019 ) | 0.016 | 0.469 | -0.1 |
| Ferritin^**^, µg/L – median (CI) | 69 (64, 76) | 48 (42, 60) | 0.0022 ( -0.042 to 0.047 ) | 0.023 | 0.922 | -0.2 |
| Vitamin B_12_^†^, pmol/L – mean (CI) | 443.97 (426.58, 461.36) | 419.66 (385.34, 453.97) | -0.0035 ( -0.013 to 0.0056 ) | 0.005 | 0.450 | -0.1 |
| Vitamin D^**^, - median (CI) | 52 (49, 55) | 55 (48, 59) | 0.12 ( 0.045 to 0.2 ) | 0.039 | 0.002 | 1.9 |
| HbA1c^**^, mean (CI) | 34.0 (33.6, 34.3) | 31.9 (31.2, 32.5) | -0.16 ( -0.5 to 0.19 ) | 0.176 | 0.372 | 0.05 |
| Immunological markers |  |  |  |  |  |  |
| Blood Leukocyte count^**^, 10^9^ cells/L - mean (CI) | 18.0 (17.6, 18.4) | 17.7 (17.0, 18.4) | 0.075 ( -0.064 to 0.21 ) | 0.070 | 0.290 | 0.03 |
| Blood Lymphocyte count, 10^9^ cells/L - mean (CI) | 2.1 (2.1, 2.2) | 2.1 (1.9, 2.2) | -0.017 ( -0.075 to 0.04 ) | 0.029 | 0.558 | -0.2 |
| Blood Monocyte count^**^, 10^9^ cells/L - mean (CI) | 0.45 (0.44, 0.47) | 0.42 (0.39, 0.45) | 0.039 ( -0.061 to 0.14 ) | 0.051 | 0.447 | -0.1 |
| Blood Neutrophil count^**^, 10^9^ cells/L - mean (CI) | 3.2 (3.0, 3.3) | 3.1 (2.8, 3.3) | 0.11 ( 0.016 to 0.2 ) | 0.047 | 0.021 | 1.0 |
| Neutrophil-to-Lymphocyte ratio^**^ – mean (CI) | 1.6 (1.5, 1.6) | 1.6 (1.4, 1.7) | 0.1 ( 0.02 to 0.18 ) | 0.041 | 0.015 | 1.1 |
| Systemic immune-inflammation index ^d**^- median (CI) | 410.8 (389.1, 432.5) | 395.8 (357.9, 433.8) | 0.095 ( 0.023 to 0.17 ) | 0.036 | 0.010 | 1.3 |
| Plasma total IgA, g/L - mean (CI) | 1.71 (1.63, 1.78) | 1.58 (1.42, 1.74) | 0.0011 ( -0.044 to 0.046 ) | 0.023 | 0.963 | -0.2 |
| Plasma total IgM^†^, g/L - mean (CI) | 1.27 (1.22, 1.33) | 1.10 (0.99, 1.22) | 0.091 ( -0.059 to 0.24 ) | 0.076 | 0.234 | 0.1 |
| Plasma total IgG^†^, g/L - mean (CI) | 11.1 (10.8, 11.3) | 10.7 (10.3, 11.1) | 0.049 ( -0.051 to 0.15 ) | 0.051 | 0.339 | 0.02 |
| hsCRP^**^, mg/L – median (CI) | 0.83 (0.73, 1.10) | 1.29 (0.74, 1.69) | 0.0054 ( -0.021 to 0.032 ) | 0.014 | 0.690 | -0.2 |
| Plasma IL-1β^**^, pg/mL – median (CI) | 0.63 (0.47, 0.73) | 0.01 (0.01, 0.19) | -0.0024 ( -0.016 to 0.011 ) | 0.007 | 0.718 | -0.2 |
| Plasma IL-2^‡,^ pg/mL - median (CI) | 0.69 (0.47, 1.09) | 0.40 (0.03, .78) | 0.045 ( -0.022 to 0.11 ) | 0.034 | 0.189 | 0.2 |
| Plasma IL-4, pg/mL - median (CI) | 1.46 (1.39, 1.50) | 0.88 (0.75, 0.92) | -0.045 ( -0.089 to -0.0019 ) | 0.022 | 0.041 | 0.7 |
| Plasma IL-7^†^, pg/mL - median (CI) | 12.6 (11.5, 12.6) | 2.98 (1.79, 5.65) | -0.023 ( -0.045 to -0.00037 ) | 0.011 | 0.046 | 0.7 |
| Plasma IL-8^‡^, pg/mL - median (CI) | 0.80 (0.58, 1.08) | 0.098 (0.077, 0.12) | -0.032 ( -0.1 to 0.04 ) | 0.037 | 0.389 | -0.1 |
| Plasma IL-9^†^, pg/mL - median (CI) | 68.2 (60.5, 80.7) | 70.2 (51.5, 86.4) | 0.00014 ( -0.0058 to 0.0061 ) | 0.003 | 0.962 | -0.2 |
| Plasma IL-12^†^, pg/mL - median (CI) | 1.49 (1.38, 1.50) | 0.194 (0.138, 1.05) | -0.011 ( -0.042 to 0.021 ) | 0.016 | 0.517 | -0.1 |
| Plasma IL-13^**^, pg/mL - median (CI) | 0.26 (0.25, 0.27) | 0.51 (0.45, 0.66) | 0.00067 ( -0.015 to 0.017 ) | 0.008 | 0.935 | -0.2 |
| Plasma IL-17A^**^, pg/mL - median (CI) | 1.62 (1.55, 1.99) | 1.35 (0.69, 2.03) | 0.0033 ( -0.019 to 0.025 ) | 0.011 | 0.766 | -0.2 |
| Plasma TNF, pg/mL - median (CI) | 7.81 (6.73, 8.24) | 4.26 (3.04, 5.40) | -0.0019 ( -0.0079 to 0.0041 ) | 0.003 | 0.531 | -0.1 |
| Plasma IFN-γ^**^, pg/mL - median (CI) | 1.30 (1.02, 1.34) | 0.94 (0.94, 1.14) | 0.026 ( 0.0029 to 0.049 ) | 0.012 | 0.028 | 0.9 |
| MCP-1/CCL2, pg/mL – mean (CI) | 12.84 (12.20, 13.47) | 13.80 (12.56, 15.04) | -0.0045 ( -0.01 to 0.0012 ) | 0.003 | 0.122 | 0.3 |
| IP-10^**^, pg/mL – mean (CI) | 164.14 (155.32, 172.96) | 125.93 (113.88, 137.99) | -0.013 ( -0.097 to 0.071 ) | 0.043 | 0.760 | -0.2 |
| Plasma Eotaxin-1/CCL11^**^, pg/mL - median (CI) | 14.8 (14.0, 15.2) | 12.7 (11.6, 14.0) | -0.057 ( -0.15 to 0.034 ) | 0.046 | 0.218 | 0.1 |
| Plasma MIP-1α^†^, pg/mL - median (CI) | 0.77 (0.67, 0.82) | 0.79 (0.79, 1.02) | -0.0084 ( -0.097 to 0.08 ) | 0.045 | 0.852 | -0.2 |
| Plasma MIP-1β^†^, pg/mL - median (CI) | 24.9 (22.5, 27.3) | 25.2 (19.4, 30.0) | -0.0058 ( -0.063 to 0.051 ) | 0.029 | 0.841 | -0.2 |
| RANTES/CCL5^**^, pg/mL – median (CI) | 261.07 (234.66, 292.45) | 271.49 (221.28, 320.20) | 0.0054 ( -0.023 to 0.034 ) | 0.014 | 0.706 | -0.2 |
| Plasma GM-CSF^**^, pg/mL - median (CI) | 0.20 (0.11, 0.34) | 0.017 (0.014, 0.023) | -0.0014 ( -0.017 to 0.014 ) | 0.008 | 0.863 | -0.2 |
| Plasma bFGF^**^, pg/mL - median (CI) | 3.40 (2.72, 3.40) | 1.32 (1.08, 1.53) | 0.009 ( -0.018 to 0.036 ) | 0.014 | 0.514 | -0.1 |
| GDF15^**^, ng/mL – mean (CI) | 0.41 (0.39, 0.42) | 0.40 (0.46, 0.43) | 0.063 ( -0.043 to 0.17 ) | 0.054 | 0.243 | 0.1 |
| TCC/C5b-9^**^, CAU/mL – median (CI) | 0.18 (0.16, 0.20) | 0.003 (0.002, 0.050) | 0.012 ( -0.0034 to 0.027 ) | 0.008 | 0.127 | 0.3 |
| Plasma C3bc^†^, ng/mL - median (CI) | 3.83 (3.67, 4.11) | 2.92 (2.70, 3.15) | 0.09 ( 0.01 to 0.17 ) | 0.041 | 0.027 | 0.9 |
| EBV prior infection^e^ – no. (%) | 277 (73.5) | 51 (60.7) | 0.038 ( -0.037 to 0.11 ) | 0.038 | 0.318 | 0.0001 |
| SARS-CoV-2-Anti-RBD^**^, BAU/mL – median (CI) | 1046 (983, 1133) | 1 (1, 1) | 0.0011 ( -0.0093 to 0.011 ) | 0.005 | 0.838 | -0.2 |
| Autonomic markers |  |  |  |  |  |  |
| LF-RRI^**^, ms^2^ – median (CI) | 654 (585, 746) | 585 (467, 841) | 0.0056 ( -0.03 to 0.041 ) | 0.018 | 0.756 | -0.2 |
| HF-RRI^**^, ms^2^ – median (CI) | 784 (682, 903) | 1006 (724, 1253) | 0.013 ( -0.017 to 0.043 ) | 0.015 | 0.407 | -0.1 |
| Cognitive function tests |  |  |  |  |  |  |
| Digit span^f^, total score – median (CI) | 15.15 (14.79, 15.51) | 14.97 (14.27, 15.68) | 0.0067 ( -0.0028 to 0.016 ) | 0.005 | 0.168 | 0.2 |
| Immediate recall^g^, score 0 to 36 – median (CI) | 24.60 (24.17, 25.02) | 24.58 (23.77, 25.39) | 0.0026 ( -0.0055 to 0.011 ) | 0.004 | 0.526 | -0.1 |
| Delayed recall^g^, score 0 to 12 – median (CI) | 8.73 (8.52, 8.94) | 8.45 (8.06, 8.84) | 0.02 ( 0.0037 to 0.037 ) | 0.009 | 0.017 | 1.0 |
| Recognition index^h^, score 0 to 12 – median (CI) | 12 (11, 12) | 12 (11, 12) | 0.017 ( -0.018 to 0.053 ) | 0.018 | 0.344 | 0.02 |
| Clinical symptoms |  |  |  |  |  |  |
| Fatigue^i§^, score 0 to 33 – mean (CI) | 16.15 (15.57, 16.74) | 13.26 (12.22, 14.31) | 0.0057 ( 0.0049 to 0.0066 ) | 0.000 | <0.001 | 27.5 |
| Post-exertional malaise^j^, score 0 to 100 – median (CI) | 20 (15, 25) | 10 (10, 15) | 0.0063 ( 0.0051 to 0.0075 ) | 0.001 | <0.001 | 18.8 |
| Sleep problems^k^, score 1 to 6 – mean (CI) | 4.05 (3.93, 4.17) | 3.83 (3.64, 4.02) | -0.15 ( -0.18 to -0.13 ) | 0.014 | <0.001 | 21.8 |
| Pain^l^, score 1 to 10 – median (CI) | 2.25 (2.00, 2.50) | 2.50 (2.00, 2.75) | 0.075 ( 0.049 to 0.1 ) | 0.013 | <0.001 | 6.6 |
| Cognitive symptoms^m^, score 3 to 15 – median (CI) | 6 (5, 6) | 6 (5, 8) | 0.049 ( 0.039 to 0.059 ) | 0.005 | <0.001 | 17.6 |
| Respiratory symptoms^n^, score 2 to 10 – median (CI) | 4 (4, 5) | 3 (3, 3) | 0.047 ( 0.031 to 0.063 ) | 0.008 | <0.001 | 6.8 |
| Autonomic symptoms^o^, score 2 to 10 - median (CI) | 6 (5, 6) | 5 (5, 6) | 0.051 ( 0.04 to 0.063 ) | 0.006 | <0.001 | 15.6 |
| Symptoms of anxiety^p^, score 0 to 21 – median (CI) | 5 (5, 6) | 7 (6, 8) | 0.032 ( 0.025 to 0.04 ) | 0.004 | <0.001 | 13.3 |
| Symptoms of depression^p^, score 0 to 21 – median (CI) | 3 (3, 4) | 3 (3, 5) | 0.032 ( 0.023 to 0.04 ) | 0.004 | <0.001 | 10.5 |
| Negative emotions^q^, score 5 to 25 – median (CI) | 9 (9, 10) | 11 (10, 13) | 0.024 ( 0.018 to 0.031 ) | 0.003 | <0.001 | 10.9 |
| Principal Component: Symptom severity^r^ | NA | NA | 0.085 ( 0.073 to 0.098 ) | 0.006 | <0.001 | 28.2 |
| Psychological traits |  |  |  |  |  |  |
| Neuroticism^s^, score 0 to 24 – median (CI) | 6 (5, 7) | 7 (5, 11) | 0.021 ( 0.016 to 0.026 ) | 0.003 | <0.001 | 12.2 |
| Emotional awareness^t^, score 7 to 35 – median (CI) | 13 (12, 14) | 14.5 (12, 17) | 0.018 ( 0.013 to 0.023 ) | 0.002 | <0.001 | 10.1 |
| Worrying tendencies^u§^, score 16 to 80 – mean (CI) | 45.03 (43.58, 46.48) | 47.74 (47.64, 47.50) | 0.00086 ( 0.00063 to 0.0011 ) | 0.000 | <0.001 | 11.2 |
| Body vigilance^v^, score 0 to 40 – mean (CI) | 12.01 (11.24, 12.79) | 11.90 (11.72, 10.83) | 0.015 ( 0.011 to 0.02 ) | 0.002 | <0.001 | 9.2 |
| Principal component: Emotional maladjustment^w^ – mean (CI) | NA | NA | -0.091 ( -0.11 to -0.072 ) | 0.010 | <0.001 | 16.0 |
| Social/behavioural markers |  |  |  |  |  |  |
| Average level of physical activity prior to acute infection^x^, score 1 to 10 – mean (CI) | 6.42 (6.20, 6.65) | 6.17 (5.72, 6.61) | -0.022 ( -0.037 to -0.0063 ) | 0.008 | 0.006 | 1.5 |
| Socioeconomic level ISEI-08^y^, score 10 to 90 – median (CI) | 63.88 (58.77, 68.54) | 63.03 (58.77, 68.70) | 0.00081 ( -9e-04 to 0.0025 ) | 0.001 | 0.352 | 0.03 |
| Family member with chronic disease^z^ – no. (%) | 123 (33.5) | 30 (35.7) | 0.095 ( 0.024 to 0.17 ) | 0.036 | 0.009 | 1.3 |
| Loneliness^aa^, score 20-80 – mean (CI) | 37.65 (36.57, 38.73) | 39.39 (36.94, 41.85) | 0.011 ( 0.008 to 0.014 ) | 0.002 | <0.001 | 10.2 |
| Negative life events last 12 months^ab^, impact score – median (CI) | 2 (1, 2) | 2 (2, 3) | 0.014 ( 0.0063 to 0.022 ) | 0.004 | <0.001 | 2.5 |
| Negative life events prior to last 12 months^ab^, impact score – median (CI) | 0 (0, 1) | 2 (0, 3) | 0.028 ( 0.013 to 0.043 ) | 0.008 | <0.001 | 2.7 |
| CI=95% Confidence interval; NA=Not applicable; SARS-CoV-2= Severe acute respiratory syndrome coronavirus 2; BMI=Body mass index; FVC=Forced vital capacity; SpO_2_=Peripheral oxygen saturation; NT-pBNP=N-terminal pro-brain natriuretic peptide; NfL=Neurofilament light chain; GFAp=Glial fibrillary acidic protein; hsCRP=high-sensitive assay of C-reactive protein; GDF-15=Growth/differentiation factor 15; IL=Interleukin; TCC=Terminal complement complex; CAU=Complement arbitrary units; RANTES=Regulated on activation, normal T-cell expressed and secreted; MCP=Monocyte chemotactic protein; IP=Interferon gamma-induced protein; RBD= Receptor binding domain; BAU=Binding antibody units; LF-RRI=Low frequency power of heart rate variability; HF-RRI=High-frequency power of heart rate variability; ISEI-08=International Socioeconomic Index 2008. ^a^ As measured by the Chalder Fatigue Questionnaire, score 0-33, higher scores imply more fatigue. Log_e_(x+1) transformation was used for regression analyses. ^a^Standardised score calculated according to World Health Organisation 2006 Child Growth Standards for ages 12-19; for participants above this age, reference values for 19-year-olds were used. ^b^One or more doses of immunisation against SARS-CoV-2. ^c^The Global Lung Function Initiative 2012 reference values were used to calculate predicted values. ^d^Defined as (NxP)/L, where N, P and L represent neutrophil, platelet and lymphocyte counts respectively. ^e^Positive IgG antibodies (VCA and/or EBNA) and negative heterophile antibodies at baseline and six months. ^f^From the Wechsler Intelligence Scales for Children revised; higher score implies better short-term memory. ^g^From the Hopkins Verbal Learning Test revised (HVLT-R); higher scores imply better immediate and delayed recall of words, respectively. ^h^From the HVLT-R; higher score implies better recognition of words. ^i^From the Chalder Fatigue Questionnaire, range 0-33; higher score implies more fatigue. ^j^From the DePaul Symptom Questionnaire; higher score implies more post-exertional malaise. ^k^From the Karolinska Sleep Questionnaire; higher score implies better sleep. ^l^From the Brief Pain Inventory, higher score implies more pain. ^m^Self-developed, aggregated score for problems with ‘memory’, ‘concentration’, and ‘decision making’; higher score implies more symptoms. ^n^Self-developed, aggregated score for symptoms ‘cough’ and ‘dyspnoea’; higher score implies more symptoms. ^o^Self-developed, aggregated score for symptoms ‘dizziness’, ‘cold and pale hands’, ‘feeling alternately warm and cold’; higher score implies more symptoms. ^p^From the anxiety and depression subscales, respectively, of the Hospital Anxiety and Depression Scale; higher scores imply more symptoms. ^q^From the Positive and Negative Affect Schedule; higher score implies more negative emotions. ^r^The main component extracted by Principal Component Analysis of the 10 clinical symptoms variables, labelled ‘symptom severity’. ^s^From the NEO-Five-Factor-Inventory-30; higher scores implies more neuroticism. ^t^From the Toronto Alexithymia Scale; higher score implies more difficulty identifying feelings. ^u^From the Penn State Worry Questionnaire; higher score implies more worrying. ^v^From the Body Vigilance Scale; higher score implies being more attentive to bodily sensations. ^w^The main component extracted by Principal Component Analysis of the four psychological traits variables, labelled ‘emotional maladjustment’. ^x^Self-developed; higher score implies more physical activity. ^y^The ISEI-08 score of the parent with the highest score; higher score implies higher socioeconomic status. ^z^Having a sibling or parent affected by chronic disease. ^aa^From the University of California, Los Angeles, Loneliness Scale; higher score implies more loneliness. ^ab^From the Life Event Checklist; higher score implies more negative impact of past life events.  Following transformations were applied to the respective variables for regression analyses: ^*^ln(x+1). ^**^ Natural logarithm. ^†^Square-root. ^‡^ Cube root § Fifth root | | | | | | |

| Table S5. Baseline predictors and their univariate associations (linear regression) to fatigue severity^*^ at 6 month follow-up. Sensitivity analysis removing individuals with possible EBV-infection at inclusion or during the observational period, vaccinated before baseline, receiving vaccination less than five days prior to the six months assessment, with possibly confounding comorbidities^#^, or with depression score at baseline above 15. | | | | |  |
| --- | --- | --- | --- | --- | --- |
|  | **Univariate association to fatigue severity (n=421)** | | | |  |
|  | *Coefficent (CI)* | *SE* | p-value | *Adjusted R^2^(%)* | *N* |
| SARS-CoV-2 status |  |  |  |  |  |
| SARS-CoV-2-positive at baseline – no. (%) | 0.072 ( -0.016 to 0.16 ) | 0.045 | 0.109 | 0.4 | 421 |
| Background and constitutional factors |  |  |  |  |  |
| Female sex – no. (%) | 0.22 ( 0.15 to 0.28 ) | 0.033 | <.001 | 8.8 | 421 |
| Age, years – mean (CI) | 0.017 ( 0.0078 to 0.026 ) | 0.005 | <.001 | 2.8 | 421 |
| BMI, z-score^a^  – mean (CI) | 0.0036 ( -0.026 to 0.033 ) | 0.015 | 0.813 | -0.2 | 420 |
| Ethnicity non-European – no. (%) | -0.021 ( -0.11 to 0.065 ) | 0.044 | 0.628 | -0.2 | 421 |
| Asthma – no.(%) | 0.12 ( -0.013 to 0.25 ) | 0.067 | 0.076 | 0.5 | 407 |
| Any comorbidity – no. (%) | 0.078 ( -0.0052 to 0.16 ) | 0.042 | 0.066 | 0.6 | 408 |
| Observational period characteristics |  |  |  |  |  |
| Time span between baseline and follow-up, days – median (range) | 0.00079 ( -0.00093 to 0.0025 ) | 0.001 | 0.367 | 0.0 | 421 |
| Immunisation against SARS-CoV-2^b^ – no. (%) | NA |  |  |  |  |
| Organ function tests/biomarkers |  |  |  |  |  |
| FVC, % of predicted^c^ – mean (CI) | -0.0014 ( -0.0048 to 0.0019 ) | 0.002 | 0.390 | -0.1 | 361 |
| SpO_2_, % – mean (CI) | 0.012 ( -0.019 to 0.042 ) | 0.016 | 0.444 | -0.1 | 419 |
| Systolic blood pressure, mmHg – mean (CI) | -0.00093 ( -0.0041 to 0.0022 ) | 0.002 | 0.564 | -0.2 | 418 |
| Diastolic blood pressure | 0.00079 ( -0.004 to 0.0056 ) | 0.002 | 0.745 | -0.2 | 418 |
| NT-pBNP^**^, ng/L – median (CI) | 0.041 ( -0.0052 to 0.087 ) | 0.023 | 0.082 | 0.5 | 394 |
| Troponin T, ng/L – median (CI) | -0.072 ( -0.16 to 0.016 ) | 0.045 | 0.108 | 0.4 | 401 |
| NfL^**^, pg/mL – mean (CI) | -0.027 ( -0.11 to 0.058 ) | 0.043 | 0.530 | -0.1 | 415 |
| GFAp^**^, pg/mL – mean (CI) | -0.038 ( -0.11 to 0.035 ) | 0.037 | 0.309 | 0.0 | 415 |
| D-dimer^**^, mg/L – median (CI) | -0.0052 ( -0.036 to 0.025 ) | 0.015 | 0.739 | -0.2 | 411 |
| Ferritin^**^, µg/L – median (CI) | 0.0096 ( -0.035 to 0.054 ) | 0.023 | 0.671 | -0.2 | 393 |
| Vitamin B_12_^†^, pmol/L – mean (CI) | -0.0018 ( -0.011 to 0.0073 ) | 0.005 | 0.698 | -0.2 | 398 |
| Vitamin D^**^, - median (CI) | 0.11 ( 0.032 to 0.19 ) | 0.039 | 0.006 | 1.6 | 410 |
| HbA1c^**^, mean (CI) | -0.13 ( -0.48 to 0.22 ) | 0.177 | 0.458 | -0.1 | 398 |
| Immunological markers |  |  |  |  |  |
| Blood Leukocyte count^**^, 10^9^ cells/L - mean (CI) | 0.082 ( -0.054 to 0.22 ) | 0.069 | 0.237 | 0.1 | 384 |
| Blood Lymphocyte count, 10^9^ cells/L - mean (CI) | -0.0091 ( -0.066 to 0.048 ) | 0.029 | 0.755 | -0.2 | 394 |
| Blood Monocyte count^**^, 10^9^ cells/L - mean (CI) | 0.048 ( -0.052 to 0.15 ) | 0.051 | 0.350 | 0.0 | 395 |
| Blood Neutrophil count^**^, 10^9^ cells/L - mean (CI) | 0.11 ( 0.019 to 0.2 ) | 0.046 | 0.018 | 1.2 | 394 |
| Neutrophil-to-Lymphocyte ratio^**^ – mean (CI) | 0.097 ( 0.017 to 0.18 ) | 0.040 | 0.017 | 1.2 | 394 |
| Systemic immune-inflammation index ^d**^- median (CI) | 0.095 ( 0.023 to 0.17 ) | 0.036 | 0.009 | 1.5 | 385 |
| Plasma total IgA, g/L - mean (CI) | 0.0099 ( -0.035 to 0.055 ) | 0.023 | 0.668 | -0.2 | 406 |
| Plasma total IgM^†^, g/L - mean (CI) | 0.12 ( -0.031 to 0.27 ) | 0.077 | 0.118 | 0.4 | 407 |
| Plasma total IgG^†^, g/L - mean (CI) | 0.077 ( -0.022 to 0.18 ) | 0.050 | 0.129 | 0.3 | 406 |
| hsCRP^**^, mg/L – median (CI) | 0.0077 ( -0.019 to 0.034 ) | 0.014 | 0.570 | -0.2 | 407 |
| Plasma IL-1β^**^, pg/mL – median (CI) | -0.00077 ( -0.014 to 0.013 ) | 0.007 | 0.909 | -0.2 | 407 |
| Plasma IL-2^‡,^ pg/mL - median (CI) | 0.053 ( -0.015 to 0.12 ) | 0.035 | 0.129 | 0.3 | 407 |
| Plasma IL-4, pg/mL - median (CI) | -0.029 ( -0.073 to 0.016 ) | 0.023 | 0.204 | 0.2 | 407 |
| Plasma IL-7^†^, pg/mL - median (CI) | -0.019 ( -0.042 to 0.0031 ) | 0.011 | 0.092 | 0.5 | 407 |
| Plasma IL-8^‡^, pg/mL - median (CI) | -0.03 ( -0.1 to 0.042 ) | 0.037 | 0.409 | -0.1 | 407 |
| Plasma IL-9^†^, pg/mL - median (CI) | -0.00086 ( -0.0068 to 0.005 ) | 0.003 | 0.774 | -0.2 | 407 |
| Plasma IL-12^†^, pg/mL - median (CI) | -0.0089 ( -0.041 to 0.023 ) | 0.016 | 0.587 | -0.2 | 407 |
| Plasma IL-13^**^, pg/mL - median (CI) | -0.0041 ( -0.02 to 0.012 ) | 0.008 | 0.620 | -0.2 | 407 |
| Plasma IL-17A^**^, pg/mL - median (CI) | 0.002 ( -0.02 to 0.024 ) | 0.011 | 0.856 | -0.2 | 407 |
| Plasma TNF, pg/mL - median (CI) | -0.00074 ( -0.0068 to 0.0053 ) | 0.003 | 0.811 | -0.2 | 407 |
| Plasma IFN-γ^**^, pg/mL - median (CI) | 0.021 ( -0.0029 to 0.045 ) | 0.012 | 0.085 | 0.5 | 407 |
| MCP-1/CCL2, pg/mL – mean (CI) | -0.0027 ( -0.0083 to 0.0029 ) | 0.003 | 0.346 | 0.0 | 407 |
| IP-10^**^, pg/mL – mean (CI) | -0.011 ( -0.098 to 0.075 ) | 0.044 | 0.799 | -0.2 | 407 |
| Plasma Eotaxin-1/CCL11^**^, pg/mL - median (CI) | -0.016 ( -0.11 to 0.077 ) | 0.047 | 0.739 | -0.2 | 407 |
| Plasma MIP-1α^†^, pg/mL - median (CI) | -0.02 ( -0.11 to 0.068 ) | 0.044 | 0.658 | -0.2 | 407 |
| Plasma MIP-1β^†^, pg/mL - median (CI) | -0.014 ( -0.071 to 0.042 ) | 0.029 | 0.621 | -0.2 | 407 |
| RANTES/CCL5^**^, pg/mL – median (CI) | 0.0012 ( -0.027 to 0.029 ) | 0.014 | 0.933 | -0.2 | 407 |
| Plasma GM-CSF^**^, pg/mL - median (CI) | 0.0019 ( -0.014 to 0.018 ) | 0.008 | 0.816 | -0.2 | 407 |
| Plasma bFGF^**^, pg/mL - median (CI) | 0.0096 ( -0.019 to 0.038 ) | 0.014 | 0.504 | -0.1 | 407 |
| GDF15^**^, ng/mL – mean (CI) | 0.052 ( -0.057 to 0.16 ) | 0.055 | 0.346 | 0.0 | 407 |
| TCC/C5b-9^**^, CAU/mL – median (CI) | 0.017 ( 0.0014 to 0.032 ) | 0.008 | 0.032 | 0.9 | 407 |
| Plasma C3bc^†^, ng/mL - median (CI) | 0.099 ( 0.017 to 0.18 ) | 0.042 | 0.018 | 1.1 | 407 |
| EBV prior infection^e^ – no. (%) | 0.013 ( -0.063 to 0.089 ) | 0.039 | 0.733 | -0.2 | 416 |
| SARS-CoV-2-Anti-RBD^**^, BAU/mL – median (CI) | 0.0048 ( -0.0057 to 0.015 ) | 0.005 | 0.372 | 0.0 | 415 |
| Autonomic markers |  |  |  |  |  |
| LF-RRI^**^, ms^2^ – median (CI) | -0.014 ( -0.05 to 0.021 ) | 0.018 | 0.426 | -0.1 | 418 |
| HF-RRI^**^, ms^2^ – median (CI) | 0.0059 ( -0.024 to 0.036 ) | 0.015 | 0.701 | -0.2 | 418 |
| Cognitive function tests |  |  |  |  |  |
| Digit span^f^, total score – median (CI) | 0.0052 ( -0.0045 to 0.015 ) | 0.005 | 0.293 | 0.0 | 418 |
| Immediate recall^g^, score 0 to 36 – median (CI) | 0.00077 ( -0.0073 to 0.0089 ) | 0.004 | 0.852 | -0.2 | 418 |
| Delayed recall^g^, score 0 to 12 – median (CI) | 0.022 ( 0.0057 to 0.039 ) | 0.008 | 0.009 | 1.4 | 418 |
| Recognition index^h^, score 0 to 12 – median (CI) | 0.023 ( -0.012 to 0.059 ) | 0.018 | 0.193 | 0.2 | 417 |
| Clinical symptoms |  |  |  |  |  |
| Fatigue^i§^, score 0 to 33 – mean (CI) | 0.0054 ( 0.0045 to 0.0063 ) | 0.0004 | <.001 | 26.8 | 406 |
| Post-exertional malaise^j^, score 0 to 100 – median (CI) | 0.0063 ( 0.0051 to 0.0075 ) | 0.001 | <.001 | 20.6 | 406 |
| Sleep problems^k^, score 1 to 6 – mean (CI) | -0.14 ( -0.17 to -0.12 ) | 0.014 | <.001 | 21.5 | 406 |
| Pain^l^, score 1 to 10 – median (CI) | 0.069 ( 0.042 to 0.095 ) | 0.013 | <.001 | 6.0 | 406 |
| Cognitive symptoms^m^, score 3 to 15 – median (CI) | 0.045 ( 0.035 to 0.055 ) | 0.005 | <.001 | 15.5 | 406 |
| Respiratory symptoms^n^, score 2 to 10 – median (CI) | 0.048 ( 0.032 to 0.063 ) | 0.008 | <.001 | 7.8 | 406 |
| Autonomic symptoms^o^, score 2 to 10 - median (CI) | 0.049 ( 0.039 to 0.06 ) | 0.006 | <.001 | 16.2 | 406 |
| Symptoms of anxiety^p^, score 0 to 21 – median (CI) | 0.031 ( 0.024 to 0.039 ) | 0.004 | <.001 | 13.5 | 406 |
| Symptoms of depression^p^, score 0 to 21 – median (CI) | 0.03 ( 0.022 to 0.039 ) | 0.004 | <.001 | 10.2 | 406 |
| Negative emotions^q^, score 5 to 25 – median (CI) | 0.023 ( 0.016 to 0.029 ) | 0.003 | <.001 | 10.7 | 406 |
| Principal Component: Symptom severity^r^ | 0.081 ( 0.068 to 0.093 ) | 0.006 | <.001 | 28.0 | 406 |
| Psychological traits |  |  | <.001 |  |  |
| Neuroticism^s^, score 0 to 24 – median (CI) | 0.020 ( 0.014 to 0.025 ) | 0.003 | <.001 | 11.3 | 406 |
| Emotional awareness^t^, score 7 to 35 – median (CI) | 0.017 ( 0.012 to 0.022 ) | 0.003 | <.001 | 9.3 | 406 |
| Worrying tendencies^u§^, score 16 to 80 – mean (CI) | 0.0008 ( 0.00058 to 0.001 ) | 0.0001 | <.001 | 10.7 | 406 |
| Body vigilance^v^, score 0 to 40 – mean (CI) | 0.013 ( 0.0089 to 0.018 ) | 0.002 | <.001 | 7.7 | 406 |
| Principal component: Emotional maladjustment^w^ – mean (CI) | 0.083 ( 0.064 to 0.1 ) | 0.010 |  | 14.8 | 406 |
| Social/behavioural markers |  |  | 0.028 |  |  |
| Average level of physical activity prior to acute infection^x^, score 1 to 10 – mean (CI) | -0.018 ( -0.034 to -0.002 ) | 0.008 | 0.648 | 0.9 | 406 |
| Socioeconomic level ISEI-08^y^, score 10 to 90 – median (CI) | 0.0004 ( -0.0013 to 0.0021 ) | 0.001 | 0.061 | -0.2 | 381 |
| Family member with chronic disease^z^ – no. (%) | 0.069 ( -0.0032 to 0.14 ) | 0.037 | <.001 | 0.6 | 406 |
| Loneliness^aa^, score 20-80 – mean (CI) | 0.01 ( 0.0074 to 0.014 ) | 0.002 | 0.007 | 9.6 | 406 |
| Negative life events last 12 months^ab^, impact score – median (CI) | 0.011 ( 0.0031 to 0.019 ) | 0.004 | 0.001 | 1.6 | 406 |
| Negative life events prior to last 12 months^ab^, impact score – median (CI) | 0.026 ( 0.011 to 0.042 ) | 0.008 | 0.001 | 2.5 | 406 |

CI=95% Confidence interval; NA=Not applicable; SARS-CoV-2= Severe acute respiratory syndrome coronavirus 2; BMI=Body mass index; FVC=Forced vital capacity; SpO_2_=Peripheral oxygen saturation; NT-pBNP=N-terminal pro-brain natriuretic peptide; NfL=Neurofilament light chain; GFAp=Glial fibrillary acidic protein; hsCRP=high-sensitive assay of C-reactive protein; GDF-15=Growth/differentiation factor 15; IL=Interleukin; TCC=Terminal complement complex; CAU=Complement arbitrary units; RANTES=Regulated on activation, normal T-cell expressed and secreted; MCP=Monocyte chemotactic protein; IP=Interferon gamma-induced protein; RBD= Receptor binding domain; BAU=Binding antibody units; LF-RRI=Low frequency power of heart rate variability; HF-RRI=High-frequency power of heart rate variability; ISEI-08=International Socioeconomic Index 2008. ^a^ As measured by the Chalder Fatigue Questionnaire, score 0-33, higher scores imply more fatigue. Log_e_(x+1) transformation was used for regression analyses. ^a^Standardised score calculated according to World Health Organisation 2006 Child Growth Standards for ages 12-19; for participants above this age, reference values for 19-year-olds were used. ^b^One or more doses of immunisation against SARS-CoV-2. ^c^The Global Lung Function Initiative 2012 reference values were used to calculate predicted values. ^d^Defined as (NxP)/L, where N, P and L represent neutrophil, platelet and lymphocyte counts respectively. ^e^Positive IgG antibodies (VCA and/or EBNA) and negative heterophile antibodies at baseline and six months. ^f^From the Wechsler Intelligence Scales for Children revised; higher score implies better short-term memory. ^g^From the Hopkins Verbal Learning Test revised (HVLT-R); higher scores imply better immediate and delayed recall of words, respectively. ^h^From the HVLT-R; higher score implies better recognition of words. ^i^From the Chalder Fatigue Questionnaire; higher score implies more fatigue. ^j^From the DePaul Symptom Questionnaire; higher score implies more post-exertional malaise. ^k^From the Karolinska Sleep Questionnaire; higher score implies better sleep. ^l^From the Brief Pain Inventory, higher score implies more pain. ^m^Self-developed, aggregated score for problems with ‘memory’, ‘concentration’, and ‘decision making’; higher score implies more symptoms. ^n^Self-developed, aggregated score for symptoms ‘cough’ and ‘dyspnoea’; higher score implies more symptoms. ^o^Self-developed, aggregated score for symptoms ‘dizziness’, ‘cold and pale hands’, ‘feeling alternately warm and cold’; higher score implies more symptoms. ^p^From the anxiety and depression subscales, respectively, of the Hospital Anxiety and Depression Scale; higher scores imply more symptoms. ^q^From the Positive and Negative Affect Schedule; higher score implies more negative emotions. ^r^The main component extracted by Principal Component Analysis of the 10 clinical symptoms variables, labelled ‘symptom severity’. ^s^From the NEO-Five-Factor-Inventory-30; higher scores implies more neuroticism. ^t^From the Toronto Alexithymia Scale; higher score implies more difficulty identifying feelings. ^u^From the Penn State Worry Questionnaire; higher score implies more worrying. ^v^From the Body Vigilance Scale; higher score implies being more attentive to bodily sensations. ^w^The main component extracted by Principal Component Analysis of the four psychological traits variables, labelled ‘emotional maladjustment’. ^x^Self-developed; higher score implies more physical activity. ^y^The ISEI-08 score of the parent with the highest score; higher score implies higher socioeconomic status. ^z^Having a sibling or parent affected by chronic disease. ^aa^From the University of California, Los Angeles, Loneliness Scale; higher score implies more loneliness. ^ab^From the Life Event Checklist; higher score implies more negative impact of past life events.

Following transformations were applied to the respective variables for regression analyses: ^*^ln(x+1). ^*^**^*^** Natural logarithm. ^†^Square-root. ^‡^ Cube root § Fifth root

# Complex chronic conditions (Feudtner et al., 2001), pain-related comorbidities (e.g. migraine) or fatigue-related comorbidities (e.g. chronic fatigue syndrome), **and** evidence of pre-existing fatigue in medical records.

| Table S6. Baseline predictors and their univariate associations (linear regression) to fatigue severity^*^ at 6-month follow-up. Sensitivity analysis featuring multiple imputation (predictive mean matching) of missing values. Pooled results from 45 imputed datasets | | | | |
| --- | --- | --- | --- | --- |
|  | **Univariate association to fatigue severity (n=467)** | | | |
|  | *Coefficent (CI)* | *SE* | p-value | *R^2^(%)* |
| SARS-CoV-2 status |  |  |  |  |
| SARS-CoV-2-positive at baseline – no. (%) | 0.057 ( -0.03 to 0.14 ) | 0.044 | 0.202 | 0.3 |
| Background and constitutional factors |  |  |  |  |
| Female sex – no. (%) | 0.23 ( 0.16 to 0.3 ) | 0.033 | <0.001 | 9.2 |
| Age, years – mean (CI) | 0.017 ( 0.0077 to 0.026 ) | 0.005 | <0.001 | 2.7 |
| BMI, z-score^a^  – mean (CI) | -0.0014 ( -0.031 to 0.028 ) | 0.015 | 0.923 | 0.0 |
| Ethnicity non-European – no. (%) | -0.034 ( -0.12 to 0.051 ) | 0.043 | 0.435 | 0.1 |
| Asthma – no.(%) | 0.081 ( -0.06 to 0.22 ) | 0.072 | 0.261 | 0.3 |
| Any comorbidity – no. (%) | 0.065 ( -0.017 to 0.15 ) | 0.041 | 0.121 | 0.6 |
| Observational period characteristics |  |  |  |  |
| Time span between baseline and follow-up, days – median (range) | 0.00023 ( -0.0015 to 0.002 ) | 0.001 | 0.799 | 0.0 |
| Immunisation against SARS-CoV-2^b^ – no. (%) | 0.26 ( -0.013 to 0.54 ) | 0.140 | 0.062 | 0.7 |
| Organ function tests/biomarkers |  |  |  |  |
| FVC, % of predicted^c^ – mean (CI) | -0.0024 ( -0.006 to 0.0013 ) | 0.002 | 0.198 | 0.4 |
| SpO_2_, % – mean (CI) | 0.018 ( -0.012 to 0.048 ) | 0.015 | 0.245 | 0.3 |
| Systolic blood pressure, mmHg – mean (CI) | -0.002 ( -0.0051 to 0.001 ) | 0.002 | 0.192 | 0.4 |
| Diastolic blood pressure | -0.00024 ( -0.005 to 0.0045 ) | 0.002 | 0.920 | 0.0 |
| NT-pBNP^**^, ng/L – median (CI) | 0.041 ( -0.0031 to 0.085 ) | 0.022 | 0.068 | 0.7 |
| Troponin T, ng/L – median (CI) | -0.089 ( -0.17 to -0.005 ) | 0.043 | 0.038 | 0.9 |
| NfL^**^, pg/mL – mean (CI) | -0.026 ( -0.11 to 0.058 ) | 0.043 | 0.543 | 0.1 |
| GFAp^**^, pg/mL – mean (CI) | -0.032 ( -0.11 to 0.041 ) | 0.037 | 0.392 | 0.2 |
| D-dimer^**^, mg/L – median (CI) | -0.011 ( -0.041 to 0.019 ) | 0.015 | 0.467 | 0.1 |
| Ferritin^**^, µg/L – median (CI) | 0.011 ( -0.039 to 0.06 ) | 0.025 | 0.676 | 0.1 |
| Vitamin B_12_^†^, pmol/L – mean (CI) | -0.0018 ( -0.011 to 0.0071 ) | 0.005 | 0.689 | 0.0 |
| Vitamin D^**^, - median (CI) | 0.12 ( 0.043 to 0.19 ) | 0.038 | 0.002 | 2.0 |
| HbA1c^**^, mean (CI) | -0.16 ( -0.49 to 0.17 ) | 0.169 | 0.350 | 0.2 |
| Immunological markers |  |  |  |  |
| Blood Leukocyte count^**^, 10^9^ cells/L - mean (CI) | 0.068 ( -0.082 to 0.22 ) | 0.076 | 0.372 | 0.2 |
| Blood Lymphocyte count, 10^9^ cells/L - mean (CI) | -0.021 ( -0.088 to 0.045 ) | 0.034 | 0.526 | 0.1 |
| Blood Monocyte count^**^, 10^9^ cells/L - mean (CI) | 0.022 ( -0.087 to 0.13 ) | 0.056 | 0.687 | 0.1 |
| Blood Neutrophil count^**^, 10^9^ cells/L - mean (CI) | 0.1 ( 0.0015 to 0.2 ) | 0.050 | 0.047 | 0.9 |
| Neutrophil-to-Lymphocyte ratio^**^ – mean (CI) | 0.096 ( 0.0093 to 0.18 ) | 0.044 | 0.030 | 1.1 |
| Systemic immune-inflammation index ^d**^- median (CI) | 0.087 ( 0.011 to 0.16 ) | 0.039 | 0.024 | 1.2 |
| Plasma total IgA, g/L - mean (CI) | 0.0013 ( -0.043 to 0.045 ) | 0.022 | 0.954 | 0.0 |
| Plasma total IgM^†^, g/L - mean (CI) | 0.088 ( -0.059 to 0.23 ) | 0.075 | 0.239 | 0.3 |
| Plasma total IgG^†^, g/L - mean (CI) | 0.054 ( -0.056 to 0.16 ) | 0.056 | 0.338 | 0.2 |
| hsCRP^**^, mg/L – median (CI) | 0.0066 ( -0.02 to 0.033 ) | 0.013 | 0.620 | 0.1 |
| Plasma IL-1β^**^, pg/mL – median (CI) | -0.0026 ( -0.016 to 0.01 ) | 0.007 | 0.689 | 0.0 |
| Plasma IL-2^‡,^ pg/mL - median (CI) | 0.044 ( -0.022 to 0.11 ) | 0.034 | 0.193 | 0.4 |
| Plasma IL-4, pg/mL - median (CI) | -0.044 ( -0.087 to -0.0018 ) | 0.022 | 0.041 | 0.9 |
| Plasma IL-7^†^, pg/mL - median (CI) | -0.022 ( -0.044 to -0.00027 ) | 0.011 | 0.047 | 0.9 |
| Plasma IL-8^‡^, pg/mL - median (CI) | -0.033 ( -0.1 to 0.038 ) | 0.036 | 0.362 | 0.2 |
| Plasma IL-9^†^, pg/mL - median (CI) | 0.00015 ( -0.0057 to 0.006 ) | 0.003 | 0.960 | 0.0 |
| Plasma IL-12^†^, pg/mL - median (CI) | -0.01 ( -0.041 to 0.021 ) | 0.016 | 0.518 | 0.1 |
| Plasma IL-13^**^, pg/mL - median (CI) | 5e-04 ( -0.015 to 0.016 ) | 0.008 | 0.949 | 0.0 |
| Plasma IL-17A^**^, pg/mL - median (CI) | 0.0035 ( -0.018 to 0.025 ) | 0.011 | 0.747 | 0.0 |
| Plasma TNF, pg/mL - median (CI) | -0.0021 ( -0.0079 to 0.0037 ) | 0.003 | 0.473 | 0.1 |
| Plasma IFN-γ^**^, pg/mL - median (CI) | 0.025 ( 0.0026 to 0.048 ) | 0.012 | 0.029 | 1.0 |
| MCP-1/CCL2, pg/mL – mean (CI) | -0.0043 ( -0.0098 to 0.0012 ) | 0.003 | 0.125 | 0.5 |
| IP-10^**^, pg/mL – mean (CI) | -0.014 ( -0.095 to 0.068 ) | 0.042 | 0.744 | 0.0 |
| Plasma Eotaxin-1/CCL11^**^, pg/mL - median (CI) | -0.056 ( -0.15 to 0.033 ) | 0.045 | 0.217 | 0.3 |
| Plasma MIP-1α^†^, pg/mL - median (CI) | -0.01 ( -0.096 to 0.076 ) | 0.044 | 0.818 | 0.0 |
| Plasma MIP-1β^†^, pg/mL - median (CI) | -0.0062 ( -0.063 to 0.05 ) | 0.029 | 0.828 | 0.0 |
| RANTES/CCL5^**^, pg/mL – median (CI) | 0.0053 ( -0.022 to 0.033 ) | 0.014 | 0.706 | 0.0 |
| Plasma GM-CSF^**^, pg/mL - median (CI) | -0.0016 ( -0.017 to 0.014 ) | 0.008 | 0.837 | 0.0 |
| Plasma bFGF^**^, pg/mL - median (CI) | 0.0079 ( -0.019 to 0.034 ) | 0.013 | 0.559 | 0.1 |
| GDF15^**^, ng/mL – mean (CI) | 0.062 ( -0.042 to 0.16 ) | 0.053 | 0.243 | 0.3 |
| TCC/C5b-9^**^, CAU/mL – median (CI) | 0.012 ( -0.0032 to 0.027 ) | 0.008 | 0.121 | 0.5 |
| Plasma C3bc^†^, ng/mL - median (CI) | 0.086 ( 0.0073 to 0.16 ) | 0.040 | 0.032 | 1.0 |
| EBV prior infection^e^ – no. (%) | 0.038 ( -0.036 to 0.11 ) | 0.038 | 0.310 | 0.2 |
| SARS-CoV-2-Anti-RBD^**^, BAU/mL – median (CI) | 0.001 ( -0.0093 to 0.011 ) | 0.005 | 0.849 | 0.0 |
| Autonomic markers |  |  |  |  |
| LF-RRI^**^, ms^2^ – median (CI) | 0.0054 ( -0.03 to 0.04 ) | 0.018 | 0.762 | 0.0 |
| HF-RRI^**^, ms^2^ – median (CI) | 0.013 ( -0.017 to 0.043 ) | 0.015 | 0.405 | 0.1 |
| Cognitive function tests |  |  |  |  |
| Digit span^f^, total score – median (CI) | 0.0067 ( -0.0029 to 0.016 ) | 0.005 | 0.170 | 0.4 |
| Immediate recall^g^, score 0 to 36 – median (CI) | 0.0025 ( -0.0056 to 0.011 ) | 0.004 | 0.537 | 0.1 |
| Delayed recall^g^, score 0 to 12 – median (CI) | 0.02 ( 0.0037 to 0.037 ) | 0.008 | 0.017 | 1.2 |
| Recognition index^h^, score 0 to 12 – median (CI) | 0.017 ( -0.019 to 0.052 ) | 0.018 | 0.356 | 0.2 |
| Clinical symptoms |  |  |  |  |
| Fatigue^i§^, score 0 to 33 – mean (CI) | 0.0057 ( 0.0048 to 0.0066 ) | 0.000 | <0.001 | 27.1 |
| Post-exertional malaise^j^, score 0 to 100 – median (CI) | 0.0062 ( 0.005 to 0.0074 ) | 0.001 | <0.001 | 18.6 |
| Sleep problems^k^, score 1 to 6 – mean (CI) | -0.15 ( -0.18 to -0.13 ) | 0.014 | <0.001 | 21.5 |
| Pain^l^, score 1 to 10 – median (CI) | 0.073 ( 0.046 to 0.099 ) | 0.013 | <0.001 | 6.3 |
| Cognitive symptoms^m^, score 3 to 15 – median (CI) | 0.049 ( 0.039 to 0.059 ) | 0.005 | <0.001 | 17.5 |
| Respiratory symptoms^n^, score 2 to 10 – median (CI) | 0.046 ( 0.03 to 0.062 ) | 0.008 | <0.001 | 6.6 |
| Autonomic symptoms^o^, score 2 to 10 - median (CI) | 0.051 ( 0.04 to 0.062 ) | 0.006 | <0.001 | 15.2 |
| Symptoms of anxiety^p^, score 0 to 21 – median (CI) | 0.032 ( 0.024 to 0.04 ) | 0.004 | <0.001 | 12.7 |
| Symptoms of depression^p^, score 0 to 21 – median (CI) | 0.031 ( 0.022 to 0.039 ) | 0.004 | <0.001 | 10.0 |
| Negative emotions^q^, score 5 to 25 – median (CI) | 0.024 ( 0.018 to 0.03 ) | 0.003 | <0.001 | 10.7 |
| Principal Component: Symptom severity^r^ | 0.085 ( 0.072 to 0.097 ) | 0.006 | <0.001 | 27.6 |
| Psychological traits |  |  |  |  |
| Neuroticism^s^, score 0 to 24 – median (CI) | 0.02 ( 0.015 to 0.026 ) | 0.003 | <0.001 | 11.5 |
| Emotional awareness^t^, score 7 to 35 – median (CI) | 0.017 ( 0.013 to 0.022 ) | 0.003 | <0.001 | 9.7 |
| Worrying tendencies^u§^, score 16 to 80 – mean (CI) | 0.00083 ( 0.00061 to 0.0011 ) | 0.000 | <0.001 | 10.6 |
| Body vigilance^v^, score 0 to 40 – mean (CI) | 0.015 ( 0.011 to 0.020 ) | 0.002 | <0.001 | 9.3 |
| Principal component: Emotional maladjustment^w^ – mean (CI) | 0.089 ( 0.070 to 0.11 ) | 0.009 | <0.001 | 15.4 |
| Social/behavioural markers |  |  |  |  |
| Average level of physical activity prior to acute infection^x^, score 1 to 10 – mean (CI) | -0.02 ( -0.035 to -0.0042 ) | 0.008 | 0.013 | 1.4 |
| Socioeconomic level ISEI-08^y^, score 10 to 90 – median (CI) | 0.00079 ( -0.001 to 0.0026 ) | 0.001 | 0.390 | 0.2 |
| Family member with chronic disease^z^ – no. (%) | 0.09 ( 0.018 to 0.16 ) | 0.037 | 0.014 | 1.3 |
| Loneliness^aa^, score 20-80 – mean (CI) | 0.011 ( 0.0077 to 0.014 ) | 0.002 | <0.001 | 9.7 |
| Negative life events last 12 months^ab^, impact score – median (CI) | 0.012 ( 0.004 to 0.021 ) | 0.004 | 0.004 | 2.1 |
| Negative life events prior to last 12 months^ab^, impact score – median (CI) | 0.028 ( 0.012 to 0.043 ) | 0.008 | <0.001 | 2.8 |
| CI=95% Confidence interval; NA=Not applicable; SARS-CoV-2= Severe acute respiratory syndrome coronavirus 2; BMI=Body mass index; FVC=Forced vital capacity; SpO_2_=Peripheral oxygen saturation; NT-pBNP=N-terminal pro-brain natriuretic peptide; NfL=Neurofilament light chain; GFAp=Glial fibrillary acidic protein; hsCRP=high-sensitive assay of C-reactive protein; GDF-15=Growth/differentiation factor 15; IL=Interleukin; TCC=Terminal complement complex; CAU=Complement arbitrary units; RANTES=Regulated on activation, normal T-cell expressed and secreted; MCP=Monocyte chemotactic protein; IP=Interferon gamma-induced protein; RBD= Receptor binding domain; BAU=Binding antibody units; LF-RRI=Low frequency power of heart rate variability; HF-RRI=High-frequency power of heart rate variability; ISEI-08=International Socioeconomic Index 2008. ^a^Standardised score calculated according to World Health Organisation 2006 Child Growth Standards for ages 12-19; for participants above this age, reference values for 19-year-olds were used. ^b^One or more doses of immunisation against SARS-CoV-2. ^c^The Global Lung Function Initiative 2012 reference values were used to calculate predicted values. ^d^Defined as (NxP)/L, where N, P and L represent neutrophil, platelet and lymphocyte counts respectively. ^e^Positive IgG antibodies (VCA and/or EBNA) and negative heterophile antibodies at baseline and six months. ^f^From the Wechsler Intelligence Scales for Children revised; higher score implies better short-term memory. ^g^From the Hopkins Verbal Learning Test revised (HVLT-R); higher scores imply better immediate and delayed recall of words, respectively. ^h^From the HVLT-R; higher score implies better recognition of words. ^i^From the Chalder Fatigue Questionnaire; higher score implies more fatigue. ^j^From the DePaul Symptom Questionnaire; higher score implies more post-exertional malaise. ^k^From the Karolinska Sleep Questionnaire; higher score implies better sleep. ^l^From the Brief Pain Inventory, higher score implies more pain. ^m^Self-developed, aggregated score for problems with ‘memory’, ‘concentration’, and ‘decision making’; higher score implies more symptoms. ^n^Self-developed, aggregated score for symptoms ‘cough’ and ‘dyspnoea’; higher score implies more symptoms. ^o^Self-developed, aggregated score for symptoms ‘dizziness’, ‘cold and pale hands’, ‘feeling alternately warm and cold’; higher score implies more symptoms. ^p^From the anxiety and depression subscales, respectively, of the Hospital Anxiety and Depression Scale; higher scores imply more symptoms. ^q^From the Positive and Negative Affect Schedule; higher score implies more negative emotions. ^r^The main component extracted by Principal Component Analysis of the 10 clinical symptoms variables, labelled ‘symptom severity’. ^s^From the NEO-Five-Factor-Inventory-30; higher scores implies more neuroticism. ^t^From the Toronto Alexithymia Scale; higher score implies more difficulty identifying feelings. ^u^From the Penn State Worry Questionnaire; higher score implies more worrying. ^v^From the Body Vigilance Scale; higher score implies being more attentive to bodily sensations. ^w^The main component extracted by Principal Component Analysis of the four psychological traits variables, labelled ‘emotional maladjustment’. ^x^Self-developed; higher score implies more physical activity. ^y^The ISEI-08 score of the parent with the highest score; higher score implies higher socioeconomic status. ^z^Having a sibling or parent affected by chronic disease. ^aa^From the University of California, Los Angeles, Loneliness Scale; higher score implies more loneliness. ^ab^From the Life Event Checklist; higher score implies more negative impact of past life events.  Following transformations were applied to the respective variables for regression analyses: ^*^ln(x+1). ^**^ Natural logarithm. ^†^Square-root. ^‡^ Cube root § Fifth root | | | | |

| Table S7. Baseline independent predictors of fatigue severity^a^ at six months follow-up. Final multiple linear regression model. Sensitivity analysis removing individuals with possible EBV-infection at inclusion or during the observational period, vaccinated before baseline, receiving vaccination less than five days prior to the six months assessment, with possibly confounding comorbidities^#^, or with depression score at baseline above 15 (n = 421). | | | | |
| --- | --- | --- | --- | --- |
|  |  | |  | |
|  | *Coefficent (CI)* | *SE* | p-value | *Delta R^2^(%)* |
| Intercept | 2.6 (2.3 to 2.9) | 0.161 | <0.001 | NA |
| SARS-CoV-2 status | 0.11 (0.034 to 0.19) |  |  |  |
| SARS-CoV-2-positive at baseline |  | 0.041 | 0.005 | 1.4 |
| Background and constitutional factors |  |  |  |  |
| Female sex | 0.13 (0.064 to 0.19) | 0.032 | 0.000 | 2.8 |
| Age, years | 0.0038 ( -0.0046 to 0.012) | 0.004 | 0.374 | 0.1 |
| BMI, z-score^b^ | 0.0021 ( -0.024 to 0.028) | 0.013 | 0.875 | 0.0 |
| Ethnicity, non-European | -0.056 ( -0.13 to 0.02) | 0.039 | 0.145 | 0.4 |
| Any comorbidity | 0.013 ( -0.06 to 0.086) | 0.037 | 0.730 | 0.02 |
| Observational period characteristics |  |  |  |  |
| Time span between baseline and follow-up, days | -0.00035 ( -0.0019 to 0.0012) | 0.001 | 0.654 | 0.04 |
| Remaining predictor variables |  |  |  |  |
| Plasma Interleukin-7^c^, pg/mL | -0.03 (-0.05 to -0.01) | 0.010 | 0.003 | 1.6 |
| Principal component: Symptom severity^d^ | 0.074 (0.061 to 0.088) | 0.007 | <0.001 | 20.8 |
|  |  |  |  |  |
| Adjusted R^2^ for the full model was 32.1%. N=392 observations.  CI=95% Confidence interval; NA=Not applicable; SARS-CoV-2= Severe acute respiratory syndrome coronavirus 2; BMI=Body mass index; . ^a^As measured by the Chalder Fatigue Questionnaire, score 0-33, higher scores imply more fatigue. Loge(x+1) transformation was used for regression analyses. ^b^Standardised score calculated according to World Health Organisation 2006 Child Growth Standards for ages 12-19; for participants above this age, reference values for 19-year-olds were used. ^c^Square-root transformation was applied before regression analyses. ^d^The main component extracted by Principal Component Analysis of the 10 clinical symptoms variables, labelled ‘symptom severity’.  # Complex chronic conditions (Feudtner et al., 2001), pain-related comorbidities (e.g. migraine) or fatigue-related comorbidities (e.g. chronic fatigue syndrome), and evidence of pre-existing fatigue in medical records. | | | | |


| Supplementary Table S8. Baseline independent predictors of fatigue severity^a^ at six months follow-up. Final multiple linear regression model. Sensitivity analysis - pooled results from 45 imputed datasets | | | | |
| --- | --- | --- | --- | --- |
|  |  | |  | |
|  | *Coefficient (CI)* | p-value | | SE |
| Intercept | 2.6 (2.3 to 2.9) | <0.001 | | 0.17 |
| SARS-CoV-2 status |  |  | |  |
| SARS-CoV-2-positive at baseline | 0.093 (0.014 to 0.17) | 0.021 | | 0.040 |
| Background and constitutional factors |  |  | |  |
| Female sex | 0.1 (0.042 to 0.16) | 0.001 | | 0.031 |
| Age, years | 0.005 (-0.0032 to 0.013) | 0.231 | | 0.0042 |
| BMI, z-score^b^ | -0.019 (-0.044 to 0.0064) | 0.142 | | 0.013 |
| Ethnicity non-European | -0.078 (-0.15 to -0.0062) | 0.033 | | 0.037 |
| Any comorbidity | -0.0093 (-0.079 to 0.061) | 0.795 | | 0.036 |
| Observational period characteristics |  |  | |  |
| Time span between baseline and follow-up, days | -0.00081 (-0.0023 to 0.00068) | 0.285 | | 0.00075 |
| Immunisation against SARS-CoV-2^c^ | 0.24 (0.0094 to 0.47) | 0.041 | | 0.12 |
| Remaining predictor variables |  |  | |  |
| Plasma Interleukin-4, pg/mL | -0.042 (-0.081 to -0.0039) | 0.031 | | 0.020 |
| Plasma Interleukin-7, pg/mL^d^ | -0.022 (-0.042 to -0.002) | 0.031 | | 0.010 |
| Plasma C3bc, ng/mL^d^ | 0.09 (0.022 to 0.16) | 0.010 | | 0.035 |
| Principal component: Symptom severity^e^ | 0.08 (0.067 to 0.093) | <0.001 | | 0.0066 |
|  |  |  | |  |
| Adjusted R^2^ for the full model was 33.6%.  CI=95% Confidence interval; NA=Not applicable; SARS-CoV-2= Severe acute respiratory syndrome coronavirus 2; BMI=Body mass index; SE = Standard error  ^a^As assessed by the Chalder Fatigue Questionnaire, score 0-33, higher scores imply more fatigue. Loge(x+1) transformation was used for regression analyses. ^b^Standardised score calculated according to World Health Organisation 2006 Child Growth Standards for ages 12-19; for participants above this age, reference values for 19-year-olds were used. ^c^One or more doses of immunisation against SARS-CoV-2. ^d^Square root-transformed variable was used for regression analyses ^e^The main component extracted by Principal Component Analysis of the 10 clinical symptoms variables, labelled ‘symptom severity’. | | | | |

| Table S9. Baseline predictors and their adjusted associations (multiple linear regression) to fatigue severity* at 6 month follow-up. Per protocol dataset. | | | | |  |
| --- | --- | --- | --- | --- | --- |
|  | **Association to fatigue severity (n=467)** | | | |  |
|  | *Coefficent (CI)* | *SE* | p-value | *Adjusted R^2^(%)* | *∆R^2^* |
| SARS-CoV-2 status |  |  |  |  |  |
| SARS-CoV-2-positive at baseline – no. (%) | 0.089 ( 0.0032 to 0.18 ) | 0.043725 | 0.042 | 10.8 | 0.6 |
| Background and constitutional factors |  |  |  |  |  |
| Female sex – no. (%) | 0.22 ( 0.15 to 0.29 ) | 0.034667 | <0.001 | 10.2 | 7.7 |
| Age, years – mean (CI) | 0.0091 ( -0.00039 to 0.019 ) | 0.004838 | 0.06 | 10.2 | 0.5 |
| BMI, z-score^a^  – mean (CI) | -0.01 ( -0.038 to 0.018 ) | 0.014216 | 0.478 | 10.2 | -0.2 |
| Ethnicity non-European – no. (%) | -0.035 ( -0.12 to 0.047 ) | 0.041946 | 0.399 | 10.2 | -0.1 |
| Asthma – no.(%) | 0.087 ( -0.061 to 0.23 ) | 0.075264 | 0.249 | 10.3 | 0.1 |
| Any comorbidity – no. (%) | 0.04 ( -0.037 to 0.12 ) | 0.038955 | 0.309 | 10.2 | 0.5 |
| Observational period characteristics |  |  |  |  |  |
| Time span between baseline and follow-up, days – median (range) | -0.00014 ( -0.0019 to 0.0016 ) | 0.000876 | 0.876 | 10.2 | -0.2 |
| Immunisation against SARS-CoV-2^b^ – no. (%) | 0.15 ( -0.11 to 0.42 ) | 0.134812 | 0.253 | 10.2 | 0.1 |
| Organ function tests/biomarkers |  |  |  |  |  |
| FVC, % of predicted^c^ – mean (CI) | -0.003 ( -0.0065 to 0.00047 ) | 0.001769 | 0.09 | 9.7 | -0.5 |
| SpO_2_, % – mean (CI) | -0.0016 ( -0.032 to 0.029 ) | 0.015614 | 0.917 | 10.0 | -0.2 |
| Systolic blood pressure, mmHg – mean (CI) | -0.00059 ( -0.004 to 0.0028 ) | 0.001727 | 0.732 | 10.1 | -0.1 |
| Diastolic blood pressure | -0.0028 ( -0.0079 to 0.0023 ) | 0.002583 | 0.275 | 10.4 | 0.2 |
| NT-pBNP^**^, ng/L – median (CI) | -0.0041 ( -0.05 to 0.042 ) | 0.023507 | 0.861 | 9.9 | -0.3 |
| Troponin T, ng/L – median (CI) | 0.031 ( -0.059 to 0.12 ) | 0.045785 | 0.501 | 9.7 | -0.5 |
| NfL^**^, pg/mL – mean (CI) | -0.027 ( -0.11 to 0.06 ) | 0.044223 | 0.54 | 9.9 | -0.3 |
| GFAp^**^, pg/mL – mean (CI) | -0.021 ( -0.095 to 0.053 ) | 0.037529 | 0.58 | 9.9 | -0.3 |
| D-dimer^**^, mg/L – median (CI) | -0.013 ( -0.042 to 0.017 ) | 0.014865 | 0.394 | 10.7 | 0.5 |
| Ferritin^**^, µg/L – median (CI) | 0.018 ( -0.029 to 0.066 ) | 0.024077 | 0.446 | 10.8 | 0.6 |
| Vitamin B_12_^†^, pmol/L – mean (CI) | 0.0032 ( -0.0058 to 0.012 ) | 0.004584 | 0.49 | 9.9 | -0.3 |
| Vitamin D^**^, - median (CI) | 0.057 ( -0.02 to 0.13 ) | 0.039115 | 0.147 | 10.3 | 0.1 |
| HbA1c^**^, mean (CI) | -0.056 ( -0.4 to 0.28 ) | 0.172892 | 0.744 | 10.0 | -0.2 |
| Immunological markers |  |  |  |  |  |
| Blood Leukocyte count^**^, 10^9^ cells/L - mean (CI) | -0.047 ( -0.19 to 0.094 ) | 0.071723 | 0.515 | 10.6 | 0.4 |
| Blood Lymphocyte count, 10^9^ cells/L - mean (CI) | -0.014 ( -0.07 to 0.043 ) | 0.028819 | 0.638 | 10.1 | -0.2 |
| Blood Monocyte count^**^, 10^9^ cells/L - mean (CI) | 0.043 ( -0.052 to 0.14 ) | 0.048611 | 0.372 | 10.2 | 0.0 |
| Blood Neutrophil count^**^, 10^9^ cells/L - mean (CI) | 8e-04 ( -0.097 to 0.098 ) | 0.049505 | 0.987 | 10.0 | -0.2 |
| Neutrophil-to-Lymphocyte ratio^**^ – mean (CI) | 0.015 ( -0.068 to 0.097 ) | 0.041963 | 0.721 | 10.0 | -0.2 |
| Systemic immune-inflammation index ^d**^- median (CI) | 0.025 ( -0.048 to 0.099 ) | 0.037228 | 0.496 | 10.4 | 0.2 |
| Plasma total IgA, g/L - mean (CI) | -0.011 ( -0.055 to 0.034 ) | 0.022716 | 0.64 | 10.4 | 0.2 |
| Plasma total IgM^†^, g/L - mean (CI) | -0.077 ( -0.23 to 0.075 ) | 0.077318 | 0.32 | 10.4 | 0.2 |
| Plasma total IgG^†^, g/L - mean (CI) | 0.016 ( -0.086 to 0.12 ) | 0.052027 | 0.76 | 10.1 | -0.1 |
| hsCRP^**^, mg/L – median (CI) | -0.00017 ( -0.031 to 0.031 ) | 0.015684 | 0.992 | 9.6 | -0.6 |
| Plasma IL-1β^**^, pg/mL – median (CI) | -0.0011 ( -0.014 to 0.012 ) | 0.00651 | 0.871 | 9.6 | -0.6 |
| Plasma IL-2^‡,^ pg/mL - median (CI) | 0.027 ( -0.039 to 0.094 ) | 0.033765 | 0.419 | 9.7 | -0.5 |
| Plasma IL-4, pg/mL - median (CI) | -0.041 ( -0.084 to 0.0023 ) | 0.021833 | 0.063 | 10.3 | 0.1 |
| Plasma IL-7^†^, pg/mL - median (CI) | -0.017 ( -0.038 to 0.0052 ) | 0.011108 | 0.135 | 10.1 | -0.1 |
| Plasma IL-8^‡^, pg/mL - median (CI) | -0.017 ( -0.086 to 0.052 ) | 0.035062 | 0.623 | 9.7 | -0.6 |
| Plasma IL-9^†^, pg/mL - median (CI) | 0.0012 ( -0.0046 to 0.0069 ) | 0.002924 | 0.684 | 9.6 | -0.6 |
| Plasma IL-12^†^, pg/mL - median (CI) | -0.006 ( -0.037 to 0.025 ) | 0.015592 | 0.703 | 9.6 | -0.6 |
| Plasma IL-13^**^, pg/mL - median (CI) | -0.00042 ( -0.016 to 0.015 ) | 0.00797 | 0.958 | 9.6 | -0.6 |
| Plasma IL-17A^**^, pg/mL - median (CI) | -0.0016 ( -0.023 to 0.02 ) | 0.01099 | 0.888 | 9.6 | -0.6 |
| Plasma TNF, pg/mL - median (CI) | 0.0011 ( -0.0047 to 0.0069 ) | 0.002955 | 0.713 | 9.6 | -0.6 |
| Plasma IFN-γ^**^, pg/mL - median (CI) | 0.024 ( 0.0018 to 0.046 ) | 0.011302 | 0.034 | 10.5 | 0.3 |
| MCP-1/CCL2, pg/mL – mean (CI) | -2e-04 ( -0.0059 to 0.0055 ) | 0.002879 | 0.944 | 9.6 | -0.6 |
| IP-10^**^, pg/mL – mean (CI) | -0.0013 ( -0.082 to 0.079 ) | 0.041081 | 0.974 | 9.6 | -0.6 |
| Plasma Eotaxin-1/CCL11^**^, pg/mL - median (CI) | -0.047 ( -0.14 to 0.045 ) | 0.04707 | 0.315 | 9.8 | -0.4 |
| Plasma MIP-1α^†^, pg/mL - median (CI) | -0.017 ( -0.1 to 0.068 ) | 0.043241 | 0.700 | 9.6 | -0.6 |
| Plasma MIP-1β^†^, pg/mL - median (CI) | 0.0069 ( -0.049 to 0.062 ) | 0.028281 | 0.808 | 9.6 | -0.6 |
| RANTES/CCL5^**^, pg/mL – median (CI) | 0.008 ( -0.019 to 0.035 ) | 0.013896 | 0.565 | 9.7 | -0.5 |
| Plasma GM-CSF^**^, pg/mL - median (CI) | 0.0044 ( -0.011 to 0.019 ) | 0.007688 | 0.571 | 9.7 | -0.5 |
| Plasma bFGF^**^, pg/mL - median (CI) | 0.0096 ( -0.017 to 0.036 ) | 0.01336 | 0.472 | 9.7 | -0.5 |
| GDF15^**^, ng/mL – mean (CI) | -0.0066 ( -0.11 to 0.097 ) | 0.052762 | 0.900 | 9.6 | -0.6 |
| TCC/C5b-9^**^, CAU/mL – median (CI) | 0.0087 ( -0.0064 to 0.024 ) | 0.0077 | 0.258 | 9.9 | -0.3 |
| Plasma C3bc^†^, ng/mL - median (CI) | 0.071 ( -0.009 to 0.15 ) | 0.040874 | 0.082 | 10.1 | -0.1 |
| EBV prior infection^e^ – no. (%) | 0.0062 ( -0.069 to 0.081 ) | 0.038132 | 0.871 | 9.9 | -0.3 |
| SARS-CoV-2-Anti-RBD^**^, BAU/mL – median (CI) | 0.006 ( -0.0041 to 0.016 ) | 0.005104 | 0.244 | 10.1 | -0.1 |
| Autonomic markers |  |  |  |  |  |
| LF-RRI^**^, ms^2^ – median (CI) | 0.018 ( -0.017 to 0.053 ) | 0.017811 | 0.312 | 10.3 | 0.1 |
| HF-RRI^**^, ms^2^ – median (CI) | 0.014 ( -0.015 to 0.043 ) | 0.014842 | 0.339 | 10.3 | 0.1 |
| Cognitive function tests |  |  |  |  |  |
| Digit span^f^, total score – median (CI) | 0.0012 ( -0.0082 to 0.011 ) | 0.004765 | 0.802 | 10.1 | -0.1 |
| Immediate recall^g^, score 0 to 36 – median (CI) | -5e-04 ( -0.0086 to 0.0076 ) | 0.004124 | 0.903 | 10.1 | -0.1 |
| Delayed recall^g^, score 0 to 12 – median (CI) | 0.013 ( -0.0047 to 0.03 ) | 0.008751 | 0.153 | 10.6 | 0.3 |
| Recognition index^h^, score 0 to 12 – median (CI) | 0.02 ( -0.014 to 0.053 ) | 0.017146 | 0.252 | 10.4 | 0.2 |
| Clinical symptoms |  |  |  |  |  |
| Fatigue^i§^, score 0 to 33 – mean (CI) | 0.0053 ( 0.0044 to 0.0062 ) | 0.000458 | <0.001 | 30.6 | 20.4 |
| Post-exertional malaise^j^, score 0 to 100 – median (CI) | 0.0056 ( 0.0044 to 0.0068 ) | 0.000624 | <0.001 | 24.0 | 13.7 |
| Sleep problems^k^, score 1 to 6 – mean (CI) | -0.13 ( -0.16 to -0.11 ) | 0.013672 | <0.001 | 26.2 | 16.0 |
| Pain^l^, score 1 to 10 – median (CI) | 0.062 ( 0.037 to 0.088 ) | 0.013002 | <0.001 | 14.5 | 4.3 |
| Cognitive symptoms^m^, score 3 to 15 – median (CI) | 0.042 ( 0.032 to 0.052 ) | 0.005012 | <0.001 | 22.5 | 12.3 |
| Respiratory symptoms^n^, score 2 to 10 – median (CI) | 0.045 ( 0.029 to 0.06 ) | 0.007852 | <0.001 | 16.2 | 6.0 |
| Autonomic symptoms^o^, score 2 to 10 - median (CI) | 0.045 ( 0.034 to 0.057 ) | 0.005791 | <0.001 | 20.9 | 10.7 |
| Symptoms of anxiety^p^, score 0 to 21 – median (CI) | 0.027 ( 0.02 to 0.035 ) | 0.003925 | <0.001 | 19.0 | 8.8 |
| Symptoms of depression^p^, score 0 to 21 – median (CI) | 0.031 ( 0.023 to 0.039 ) | 0.004224 | <0.001 | 19.8 | 9.5 |
| Negative emotions^q^, score 5 to 25 – median (CI) | 0.02 ( 0.013 to 0.026 ) | 0.003261 | <0.001 | 16.8 | 6.6 |
| Principal Component: Symptom severity^r^ | 0.078 ( 0.065 to 0.091 ) | 0.006668 | <0.001 | 31.4 | 21.2 |
| Psychological traits |  |  |  |  |  |
| Neuroticism^s^, score 0 to 24 – median (CI) | 0.016 ( 0.011 to 0.022 ) | 0.002739 | <0.001 | 16.7 | 6.5 |
| Emotional awareness^t^, score 7 to 35 – median (CI) | 0.014 ( 0.009 to 0.019 ) | 0.002532 | <0.001 | 15.8 | 5.6 |
| Worrying tendencies^u§^, score 16 to 80 – mean (CI) | 0.00061 ( 0.00037 to 0.00085 ) | 0.000122 | <0.001 | 14.8 | 4.6 |
| Body vigilance^v^, score 0 to 40 – mean (CI) | 0.012 ( 0.0076 to 0.017 ) | 0.002362 | <0.001 | 15.1 | 4.9 |
| Principal component: Emotional maladjustment^w^ – mean (CI) | -0.074 ( -0.095 to -0.053 ) | 0.010626 | <0.001 | 18.9 | 8.7 |
| Social/behavioural markers |  |  |  |  |  |
| Average level of physical activity prior to acute infection^x^, score 1 to 10 – mean (CI) | -0.014 ( -0.029 to 0.0015 ) | 0.007783 | 0.076 | 10.6 | 0.4 |
| Socioeconomic level ISEI-08^y^, score 10 to 90 – median (CI) | 0.00029 ( -0.0014 to 0.002 ) | 0.000857 | 0.736 | 10.2 | 0.0 |
| Family member with chronic disease^z^ – no. (%) | 0.051 ( -0.02 to 0.12 ) | 0.036064 | 0.158 | 10.4 | 0.2 |
| Loneliness^aa^, score 20-80 – mean (CI) | 0.0095 ( 0.0065 to 0.012 ) | 0.001516 | <0.001 | 17.3 | 7.1 |
| Negative life events last 12 months^ab^, impact score – median (CI) | 0.0098 ( 0.0021 to 0.018 ) | 0.003923 | 0.013 | 11.3 | 1.1 |
| Negative life events prior to last 12 months^ab^, impact score – median (CI) | 0.018 ( 0.0026 to 0.033 ) | 0.00765 | 0.022 | 11.1 | 0.9 |

Variables were adjusted for sex, age, BMI, comorbidities, time since baseline assessment and SARS-CoV-2 immunisation status.

CI=95% Confidence interval; NA=Not applicable; SARS-CoV-2= Severe acute respiratory syndrome coronavirus 2; BMI=Body mass index; FVC=Forced vital capacity; SpO_2_=Peripheral oxygen saturation; NT-pBNP=N-terminal pro-brain natriuretic peptide; NfL=Neurofilament light chain; GFAp=Glial fibrillary acidic protein; hsCRP=high-sensitive assay of C-reactive protein; GDF-15=Growth/differentiation factor 15; IL=Interleukin; TCC=Terminal complement complex; CAU=Complement arbitrary units; RANTES=Regulated on activation, normal T-cell expressed and secreted; MCP=Monocyte chemotactic protein; IP=Interferon gamma-induced protein; RBD= Receptor binding domain; BAU=Binding antibody units; LF-RRI=Low frequency power of heart rate variability; HF-RRI=High-frequency power of heart rate variability; ISEI-08=International Socioeconomic Index 2008. ^a^ As measured by the Chalder Fatigue Questionnaire, score 0-33, higher scores imply more fatigue. Log_e_(x+1) transformation was used for regression analyses. ^a^Standardised score calculated according to World Health Organisation 2006 Child Growth Standards for ages 12-19; for participants above this age, reference values for 19-year-olds were used. ^b^One or more doses of immunisation against SARS-CoV-2. ^c^The Global Lung Function Initiative 2012 reference values were used to calculate predicted values. ^d^Defined as (NxP)/L, where N, P and L represent neutrophil, platelet and lymphocyte counts respectively. ^e^Positive IgG antibodies (VCA and/or EBNA) and negative heterophile antibodies at baseline and six months. ^f^From the Wechsler Intelligence Scales for Children revised; higher score implies better short-term memory. ^g^From the Hopkins Verbal Learning Test revised (HVLT-R); higher scores imply better immediate and delayed recall of words, respectively. ^h^From the HVLT-R; higher score implies better recognition of words. ^i^From the Chalder Fatigue Questionnaire; higher score implies more fatigue. ^j^From the DePaul Symptom Questionnaire; higher score implies more post-exertional malaise. ^k^From the Karolinska Sleep Questionnaire; higher score implies better sleep. ^l^From the Brief Pain Inventory, higher score implies more pain. ^m^Self-developed, aggregated score for problems with ‘memory’, ‘concentration’, and ‘decision making’; higher score implies more symptoms. ^n^Self-developed, aggregated score for symptoms ‘cough’ and ‘dyspnoea’; higher score implies more symptoms. ^o^Self-developed, aggregated score for symptoms ‘dizziness’, ‘cold and pale hands’, ‘feeling alternately warm and cold’; higher score implies more symptoms. ^p^From the anxiety and depression subscales, respectively, of the Hospital Anxiety and Depression Scale; higher scores imply more symptoms. ^q^From the Positive and Negative Affect Schedule; higher score implies more negative emotions. ^r^The main component extracted by Principal Component Analysis of the 10 clinical symptoms variables, labelled ‘symptom severity’. ^s^From the NEO-Five-Factor-Inventory-30; higher scores implies more neuroticism. ^t^From the Toronto Alexithymia Scale; higher score implies more difficulty identifying feelings. ^u^From the Penn State Worry Questionnaire; higher score implies more worrying. ^v^From the Body Vigilance Scale; higher score implies being more attentive to bodily sensations. ^w^The main component extracted by Principal Component Analysis of the four psychological traits variables, labelled ‘emotional maladjustment’. ^x^Self-developed; higher score implies more physical activity. ^y^The ISEI-08 score of the parent with the highest score; higher score implies higher socioeconomic status. ^z^Having a sibling or parent affected by chronic disease. ^aa^From the University of California, Los Angeles, Loneliness Scale; higher score implies more loneliness. ^ab^From the Life Event Checklist; higher score implies more negative impact of past life events.

Following transformations were applied to the respective variables for regression analyses: *ln(x+1). ** Natural logarithm. †Square-root. ‡ Cube root § Fifth root

*
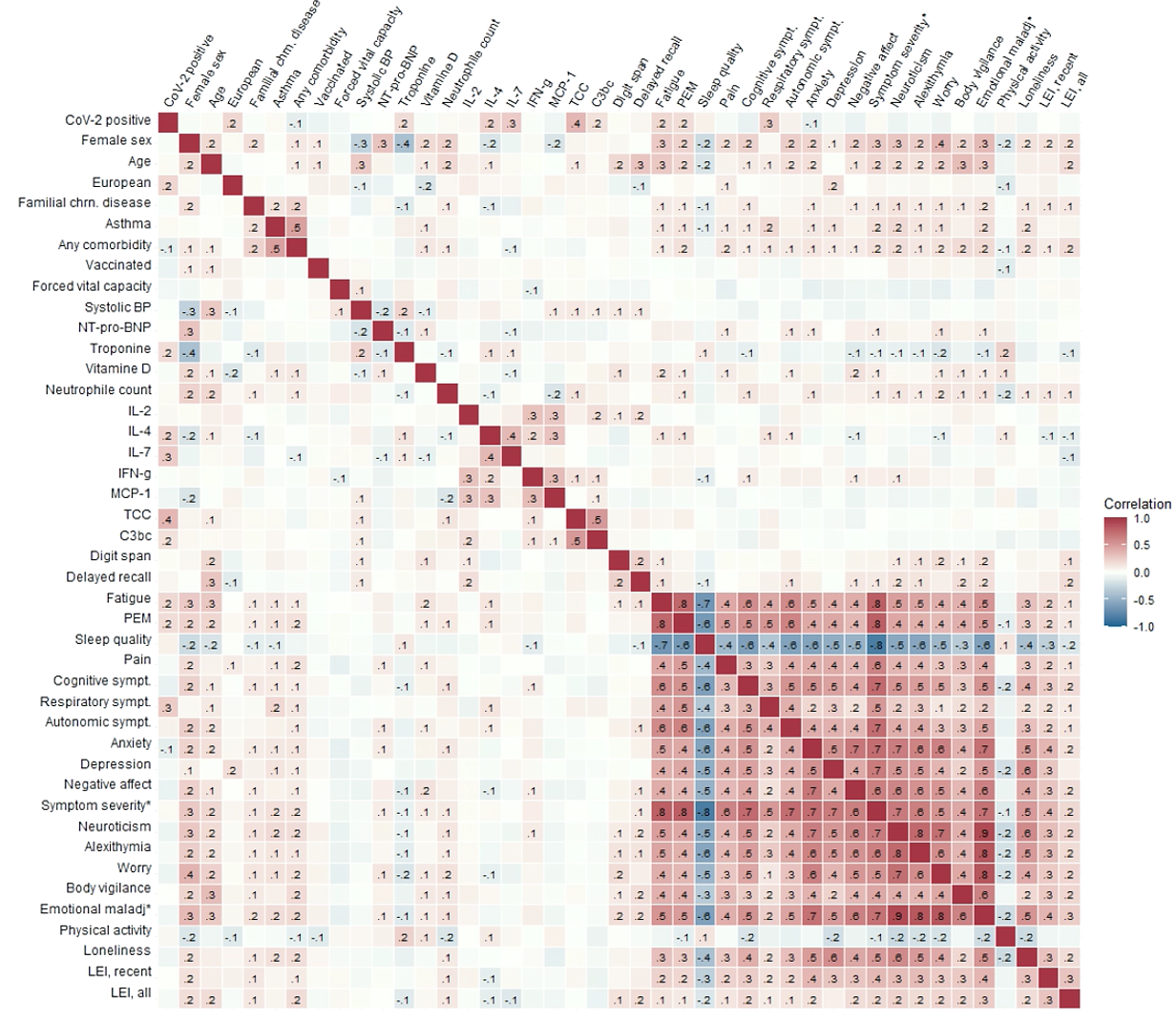
***Figure S1.** Correlation^†^ heatmap of independent predictor variables with p<0.2 in bivariate analyses. (*For clarity, correlations with p<0.5 have been omitted)*

CoV-2= Severe acute respiratory syndrome coronavirus 2; FVC=Forced vital capacity; NT-pBNP=N-terminal pro-brain natriuretic peptide; IL=Interleukin; IFN-g = Interferon gamma; MCP=Monocyte chemotactic protein; TCC=Terminal complement complext; LEI = Life Events Impact score

^†^Spearman rank correlation for continuous variables, whereas Pearson correlation was used for dichotomous variables.

*Derived respectively from the principal component of principal component analyses of “clinical symptoms” and “personality traits”-variables.

**3. References – supplementary**

1 Selvakumar J, Havdal LB, Drevvatne M, *et al.* Prevalence and Characteristics Associated With Post–COVID-19 Condition Among Nonhospitalized Adolescents and Young Adults. *JAMA Network Open* 2023; **6**: e235763.

2 Bergseth G, Ludviksen JK, Kirschfink M, Giclas PC, Nilsson B, Mollnes TE. An international serum standard for application in assays to detect human complement activation products. *Mol Immunol* 2013; **56**: 232–9.

3 Tran TT, Vaage EB, Mehta A, *et al.* Titers of antibodies the receptor-binding domain (RBD) of ancestral SARS-CoV-2 are predictive for levels of neutralizing antibodies to multiple variants. 2022; : 2022.03.26.484261.

4 Buuren S van, Groothuis-Oudshoorn K. mice: Multivariate Imputation by Chained Equations in R. *Journal of Statistical Software* 2011; **45**: 1–67.

5 Ayoubkhani D, Bermingham C, Pouwels KB, *et al.* Trajectory of long covid symptoms after covid-19 vaccination: community based cohort study. *BMJ* 2022; **377**: e069676.

6 Gherardi RK, Crépeaux G, Authier F-J. Myalgia and chronic fatigue syndrome following immunization: macrophagic myofasciitis and animal studies support linkage to aluminum adjuvant persistency and diffusion in the immune system. *Autoimmun Rev* 2019; **18**: 691–705.

7 Kennedy SH. Core symptoms of major depressive disorder: relevance to diagnosis and treatment. *Dialogues in Clinical Neuroscience* 2008; **10**: 271–7.

8 Jason LA, Evans M, Brown M, Porter N. What is Fatigue? Pathological and Nonpathological Fatigue. *PM&R* 2010; **2**: 327–31.

9 Feudtner C, Hays RM, Haynes G, Geyer JR, Neff JM, Koepsell TD. Deaths Attributed to Pediatric Complex Chronic Conditions: National Trends and Implications for Supportive Care Services. *Pediatrics* 2001; **107**: e99–e99.
